# All-optical physiology resolves a synaptic basis for behavioral timescale plasticity

**DOI:** 10.1016/j.cell.2022.12.035

**Published:** 2023-01-19

**Authors:** Linlin Z. Fan, Doo Kyung Kim, Joshua H. Jennings, He Tian, Peter Y. Wang, Charu Ramakrishnan, Sawyer Randles, Yanjun Sun, Elina Thadhani, Yoon Seok Kim, Sean Quirin, Lisa Giocomo, Adam E. Cohen, Karl Deisseroth

**Affiliations:** 1 Department of Bioengineering, Stanford University, Stanford, CA, USA; 2 Department of Chemistry and Chemical Biology, Harvard University, Cambridge, MA, USA; 3 Department of Neurobiology, Stanford University, Stanford, CA, USA; 4 Department of Physics, Harvard University, Cambridge, MA, USA; 5 Department of Psychiatry and Behavioral Sciences, Stanford University, Stanford, CA, USA; 6 Howard Hughes Medical Institute, Stanford, CA, USA; 7 Lead contact

## Abstract

Learning has been associated with modifications of synaptic and circuit properties, but the precise changes storing information in mammals have remained largely unclear. We combined genetically targeted voltage imaging with targeted optogenetic activation and silencing of pre- and post-synaptic neurons to study the mechanisms underlying hippocampal behavioral timescale plasticity. In mice navigating a virtual-reality environment, targeted optogenetic activation of individual CA1 cells at specific places induced stable representations of these places in the targeted cells. Optical elicitation, recording, and modulation of synaptic transmission in behaving mice revealed that activity in presynaptic CA2/3 cells was required for the induction of plasticity in CA1 and, furthermore, that during induction of these place fields in single CA1 cells, synaptic input from CA2/3 onto these same cells was potentiated. These results reveal synaptic implementation of hippocampal behavioral timescale plasticity and define a methodology to resolve synaptic plasticity during learning and memory in behaving mammals.

## In brief

An all-optical physiology approach is developed to study synaptic transmission and plasticity in single identified cells in behaving mammals, which reveals that presynaptic CA2/3 activity is required for CA1 plasticity and place field induction during behavior.

## Graphical Abstract

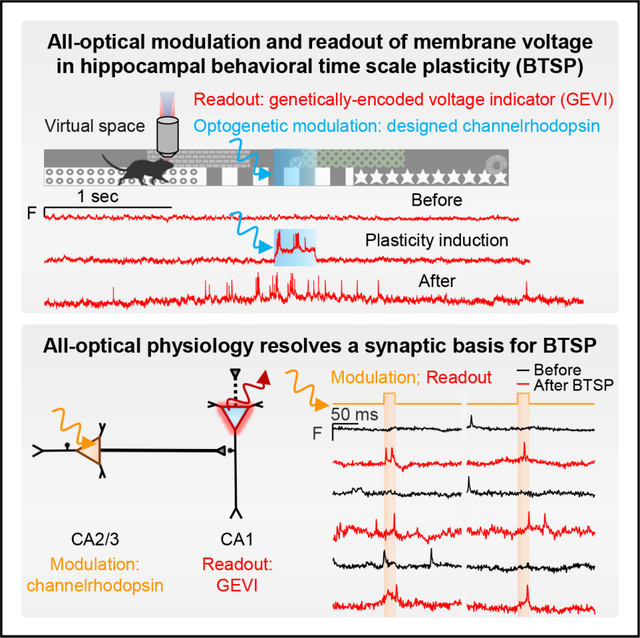

## INTRODUCTION

The specific cellular processes in the mammalian brain that implement learning remain largely unclear, although it has been hypothesized that learning-related alteration in efficacy of specific synaptic connections (synaptic plasticity) enables memory storage.^1,2^ Numerous forms of synaptic plasticity exist in mammals and many different molecular processes are involved,^3–12^ but less is understood about the timing and location of behaviorally relevant synaptic modifications during learning in behaving mammals. The hippocampus plays crucial roles in spatial and contextual memories,^13–18^ and long-term synaptic plasticity was first discovered in the hippocampus^11^; however, although many electrophysiological, genetic, and pharmacological studies have revealed correlations between hippocampal activity-dependent synaptic plasticity and learning,^15,19–21^ it has been challenging to directly and causally link activity patterns in specific pre- and post-synaptic cells with hippocampal synaptic plasticity as it manifests in behaving mammals.

Recently, a group of elegant studies used intracellular whole-cell electrophysiology to control and record the membrane potential of single hippocampal CA1 pyramidal neurons^3,22,23^ during rodent spatial navigation. Plateau potentials could rapidly elicit place field formation through a distinct form of plasticity, termed behavioral timescale synaptic plasticity (BTSP).^3^ However, since only single CA1 neurons were intracellularly accessed, it remained unknown which cellular elements were modified to give rise to this plasticity. One view had been that the feedforward synapses onto CA1 neurons from the CA3 region are potentiated under these conditions,^3,23^ but no direct evidence has been found in behaving mammals. Other studies have found that excitability mechanisms may also contribute to plasticity^24–26^; however, direct evidence distinguishing synaptic and excitability mechanisms is lacking.

To address these questions, it would be crucial to measure and manipulate synaptic transmission and plasticity involving identified cells in behaving mammals. However, this goal has long remained challenging due to difficulties with performing simultaneous electrophysiological manipulation and recording in pre- and post-synaptic cells^27,28^ and with maintaining long-term patch-clamp connections to probe long-term plasticity. All-optical approaches using Ca^2+^ imaging (together with single-cell electroporation of opsins^29,30^ or single-cell-targeted optogenetics^17,31^) cannot resolve dendritic plateau-driven complex spikes that are particularly important for synaptic plasticity,^22^ and such cellular Ca^2+^ imaging also lacks temporal and subthreshold precision sufficient to localize or quantify synaptic plasticity.

An all-optical physiology approach pairing patterned optogenetic stimulation with voltage imaging could offer the required access and precision for this goal. Recent advances with genetically encoded voltage indicators (GEVIs) and optical hardware have enabled voltage imaging of multiple cells with single-neuron, single-spike resolution *in vivo*.^32–38^ Simultaneous voltage imaging using near-infrared GEVIs based on archaerhodopsin-3^39^ and optogenetics allowed optical measurement of brain state-dependent changes in excitability^32,36^ and of excitatory and inhibitory postsynaptic potentials (IPSPs) in acute slices^40^ and awake mice,^36^ raising the possibility of optical exploration of synaptic plasticity in behaving mammals. Here, with improved optogenetic actuation and silencing tools and GEVIs,^41^ we all-optically induced and recorded hippocampal BTSP in head-fixed mice performing virtual-reality (VR) navigation,^31,42,43^ probed synaptic transmission and plasticity between projection-defined genetically targeted presynaptic CA2/3 cells and postsynaptic CA1 cells in these animals, and assessed causal role of activity in presynaptic elements for postsynaptic plasticity induction. This approach enabled resolution of synaptic processes underlying place field formation and may enable precise and causal testing of plasticity mechanisms more broadly that underly learning and memory in behaving mammals.

## RESULTS

### Voltage imaging of hippocampal dynamics during virtual-reality behavior

**Figure 1.**
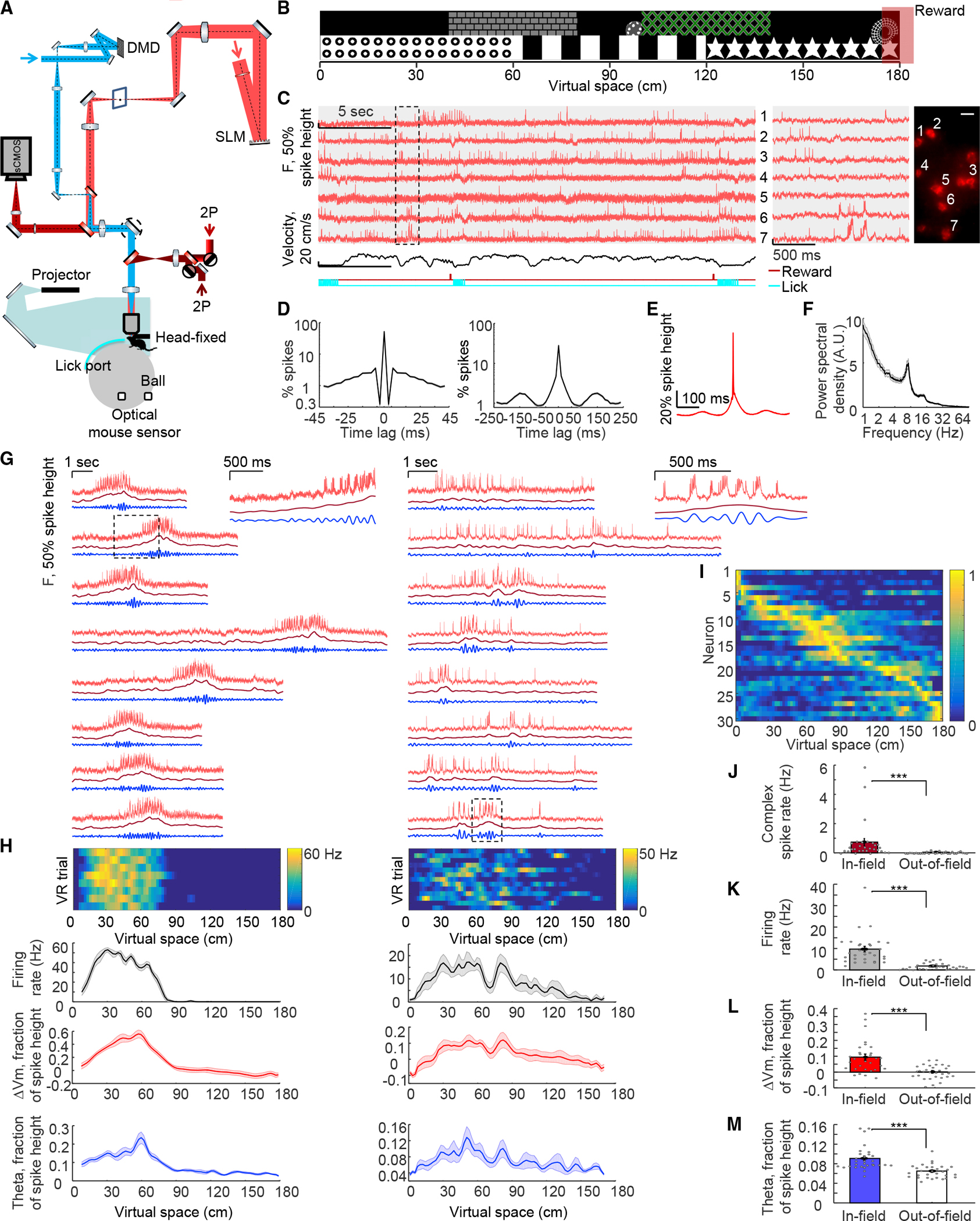
Voltage imaging of hippocampal dynamics during virtual reality (VR) behavior (A) Optical system and VR setup. Holographic structured-illumination voltage imaging (red), micromirror-patterned optogenetic stimulation (blue), and VR rig (cyan and gray). Details in STAR Methods. (B) Visual wall cues of VR environment. Red box: reward delivered at end of track on 80% of VR trials. (C) Voltage imaging of CA1 cells during VR behavior. Red: single-trial unfiltered fluorescence traces of somQuasAr6a recorded at 1 kHz. Gray shaded zones: simultaneously recorded cells. Black: mouse running velocity. Dark red line with ticks at bottom: reward delivery. Cyan: detected licking periods (reward retrieval). Middle: magnified views of boxed region at left. Right: corresponding cells showing GEVI fluorescence. Scale bars, 20 μm. (D) Spike-triggered autocorrelogram showing (left) refractory period and (right) theta oscillation (n = 117 cells, 9 mice). (E and F) (E) Spike-triggered average waveform, and (F) power spectra showing theta oscillation peaking at ~7 Hz. (G) Two representative place cells: red: GEVI fluorescence traces as a function of time for selected VR trials. Dark red: ramp-like subthreshold membrane potential (spikes removed and data low-pass filtered <3 Hz). Blue: theta oscillation (spikes removed and data bandpass filtered 6–10 Hz). Insets on the right: magnified views of the boxed regions at left. Place field size: left: 67.5 cm, right: 103.5 cm. (H) Top: firing rate of all VR trials across virtual space for the cells shown in (G). Mean firing rate maps (black), average subthreshold membrane potential changes (red), and mean theta-rhythm amplitude (blue) for the cells shown in (G). (I) Normalized firing rates of all place cells (n = 30 cells) ordered by virtual space. (J–M) (J) Mean complex spike rate, (K) mean firing rate, (L) mean subthreshold membrane potential, and (M) mean amplitude of theta-rhythm inside and outside place-field. All error bars and shading: SEM.

To achieve millivolt-scale recordings of subthreshold dynamics while minimizing in-focus and out-of-focus optical crosstalk, we built a holographic structured-illumination system^36^ and performed voltage imaging with targeted illumination of red light to excite somQuasAr6a^41^ (Figure 1A; STAR Methods). For targeted optogenetic stimulation, we used a digital micromirror device (DMD) to pattern illumination (Figure 1A; STAR Methods) and a soma-localized blue-shifted channelrhodopsin, sombC1C2TG (STAR Methods).^43–45^ In cultured neurons expressing this channelrhodopsin, blue light evoked robust photocurrents, but red light at intensities used for voltage imaging (639 nm, 2.4–9 W/mm^2^) did not (Figure S1). We created a bicistronic adenoassociated virus (AAV) for co-expression of somQuasAr6a and sombC1C2TG (Figure S1). To minimize optical crosstalk for both voltage imaging and optogenetic stimulation, we expressed this construct sparsely using a recombinase-dependent virus in CA1 and confirmed that somQuasAr6a and sombC1C2TG were restricted to the soma and proximal dendrites (Figure S1). In head-fixed mice running on a spherical treadmill, targeting optogenetic stimulation to single cells while simultaneously imaging surrounding cells revealed minimal direct stimulation of surrounding cells (Figure S1), consistent with prior results.^36^

Mice were trained to navigate in a VR system^31,42^ (Figures 1A and 1B; STAR Methods). The optical system and somQuasAr6a enabled high-resolution recordings from CA1 cells during this behavior; typical recordings (1 kHz frame rate) resolved 1–10 spiking cells simultaneously (Figure 1C). Single-trial unfiltered fluorescence traces resolved signatures of intracellular membrane potential including features of virtual-space tuning, subthreshold depolarization, and plateau-driven complex spikes (Figures 1C and S1). Simultaneously recorded cells typically showed distinct intracellular dynamics, confirming minimal optical crosstalk (Figures 1C and S1).

To estimate fidelity of optically detected spikes, we quantified signal-to-noise ratio (SNR, defined by ratio of spike height to baseline noise with 1 kHz bandwidth) and refractory period. Action potentials were recorded with SNR 8.8 ± 2.4 (mean ± SD, n=117 cells, 9 mice). For Gaussian noise, with this SNR and with spike-detection threshold set at 4σ above baseline noise, the expected false-positive rate will be <0.9 incorrectly called spikes per 30-s recording (STAR Methods); indeed, a spike-triggered autocorrelogram showed the probability of two spikes non-physiologically occurring within 3 ms of each other to be <3 × 10^−3^ (Figure 1D). False-negatives at this SNR are expected to result for <7.9 × 10^-7^ of true spikes (STAR Methods). We further used theta oscillations to validate the physiological relevance of optically detected subthreshold signals; spike-triggered average (STA) fluorescence and power spectral density, with spikes digitally removed, exhibited robust theta-rhythmicity (Figures 1D–1F).

Using standard spike-rate and spatial-information metrics,^46^ we identified 30 place cells (Figures 1G and 1I, n = 30/105 cells, 9 mice; STARMethods); these cells exhibited spatially modulated firing patterns (in-place-field: 9.7 ± 1.3 Hz, out-of-place field: 1.8 ± 0.3 Hz, mean ± SEM, n = 30 cells, p = 1 × 10^−6^, two-sided paired-sample t test, all error ranges SEM unless otherwise specified), similar to those measured using intracellular recordings^22,42^ (Figures 1H and 1K). Plateau-driven complex spikes are challenging to detect with extracellular recording^47^ or Ca^2+^ imaging; here, voltage imaging allowed detection of large long-lasting depolarizations atop spiking bursts (STAR Methods; Figure 1G), detecting 1,756 events from 104 cells. Average duration was 46 ± 1 ms (mean ± SEM, n = 1,756 events), and amplitude was 58.9% ± 0.3% of spike height; the rate of these complex spikes was greater in-field compared with out-of-field (in-field: 0.7 ± 0.2 Hz, out-of-field: 0.05 ± 0.01 Hz, p = 0.007, two-sided paired-sample t test, Figure 1J), consistent with prior results.^22,47^

Examining subthreshold dynamics of place fields, we observed ramping depolarizations in membrane potential, often preceding action-potential firing (Figure 1G). To quantify these subthreshold ramping dynamics, we removed spikes and low-pass filtered the fluorescence traces (<3 Hz; Figures 1G and 1H). Mean subthreshold depolarization was considerably greater in-field compared with out-of-field (in-field: 9.4% ± 1.8% of spike height, out-of-field: 0.1% ± 0.8%, p = 6 × 10^−6^, two-sided paired-sample t test, Figure 1L). Increased amplitude of intracellular theta-frequency oscillation (Figures 1G and 1H) inside the place field was also observed (in-field: 9.1% ± 0.4% of spike height, out-of-field: 6.5% ± 0.3%, p = 1 × 10^−5^, two-sided paired-sample t test, Figure 1M). These subthreshold signatures of place fields captured by voltage imaging were consistent with prior intracellular whole-cell recordings.^22,42,48^

### Targeted optogenetic activation recruits place-field plasticity

**Figure 2.**
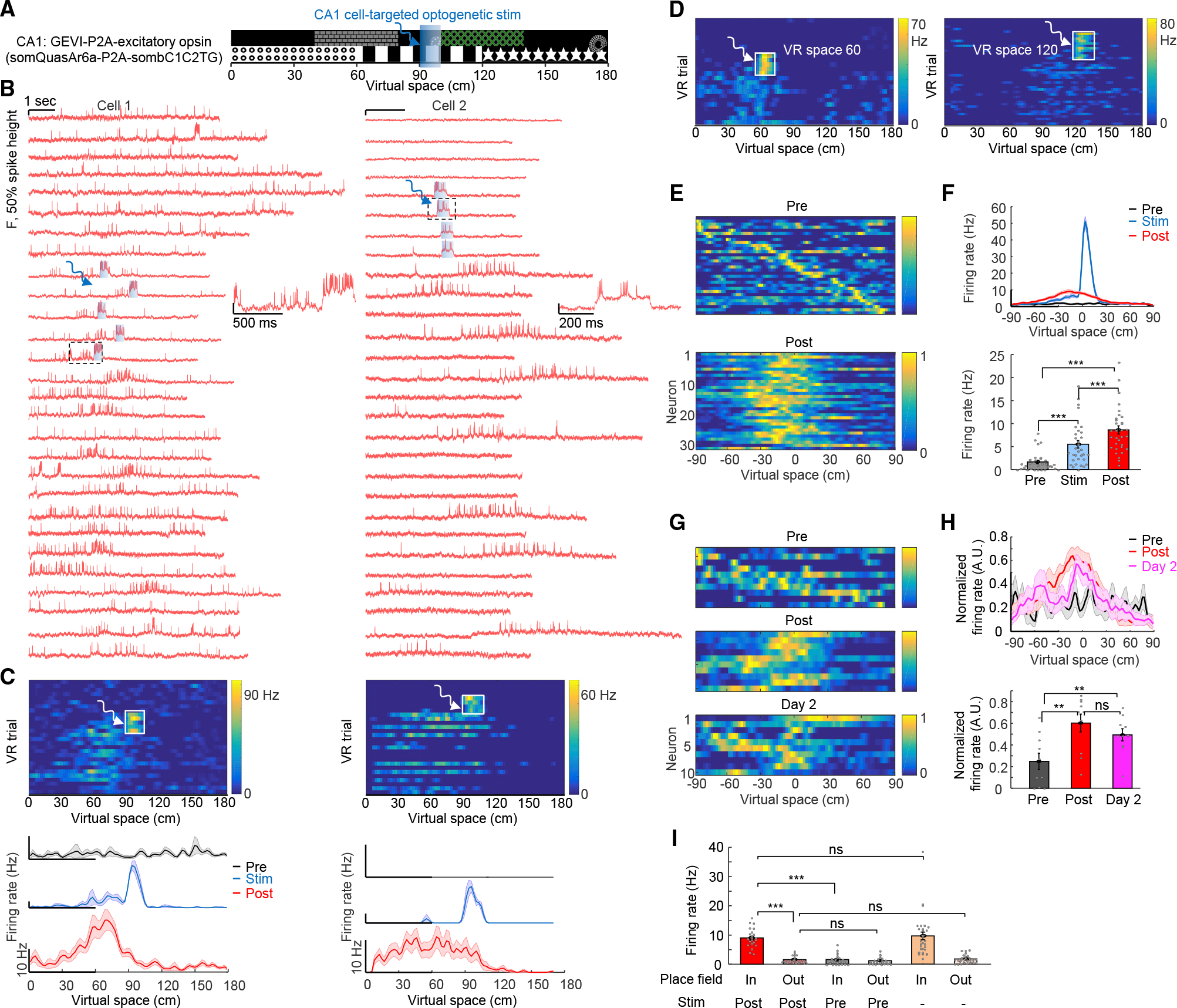
All-optical induction and recording of hippocampal BTSP (A) Closed-loop targeted optogenetic stimulation of single cells at specific locations. (B) Two example cells: fluorescence traces as a function of time for VR trials pre-, during, and post-optogenetic stimulation. Blue shaded boxes: 300 ms targeted optogenetic stimulation at 90 cm. Insets on the right: magnified views of the black dashed regions at left. Note that voltage imaging was performed by sampling through the *Pre*, *Stim,* and *Post* epochs (VR trial numbers, left: 7–8-14–15-23–24-26–27-39–40-41–49-50–51-56–57-58–62-63–64-69–70-71–77-78–84-85–86; right: 9–10-11–12-41–42-43–44-62–63-64–65-73–74-75–76-87–88-89–90-106–107-108–109-255–256-257–258). (C) Firing rate of all VR trials (pre-, during, and post-optogenetic stimulation) across virtual space for the two cells shown in (B). White: 300-ms targeted optogenetic stimulation at the 90 cm location. Bottom: average firing rate maps pre, during, and post-optogenetic stimulation for the cells shown in (B). The cell on the right was silent in the recorded VR trials before optogenetic stimulation. (D) Firing rate of all VR trials for two example cells stimulated at 60 cm (n = 5 cells) or 120 cm (n = 7 cells). (E and F) (E) Normalized firing rates, and (F) average firing rate maps of all optogenetically stimulated cells pre- and post-optogenetic stimulation (n = 32 cells). Data from different optogenetic stimulation locations aligned at 0. Bottom (F): quantification of firing rate at 10–30 cm before the optically stimulated location for *Pre, Stim*, and *Post* epochs. (G and H) (G) Normalized firing rates, and (H) average firing rate maps, of optogenetically stimulated cells pre-, post-, and 24 h (day 2) post-optogenetic stimulation (n = 10 cells). Bottom (H): firing rate at the optically stimulated location significantly increased both immediately and 24 h after optogenetic stimulation compared with pre-optogenetic stimulation. (I) For n = 25 cells passing the significance test for place cells, optogenetic stimulation did not affect out-of-field firing rate but significantly increased in-field firing rate. Optogenetically induced place cells had similar in-field and out-of-field firing rates as the natural place cells (same dataset as in Figure 1). All error bars and shading: SEM.

Intracellular sustained current injection at specific locations has been shown to rapidly induce place field formation, through generating plateau potentials that resemble naturally occurring plateau potentials.^3,22,49^ We hypothesized that targeted optogenetic stimulation could also mimic these natural processes to induce rapid place cell formation, and therefore, we performed closed-loop optogenetic stimulation at specific virtual-space locations. During each session, the animal initially ran 30 baseline trials in the virtual arena (*Pre* epoch, Figure 2A). Subsequently, we used custom VR software^50^ to deliver 300 ms optogenetic stimulation to individually identified neurons when the animal arrived at specific locations in virtual space, over 20 trials (90-cm location, *Stim* epoch, Figure 2A; STAR Methods). This was followed by test VR trials in the virtual arena, without stimulation, for another 10–30 min (*Post* epoch, Figure 2A).

We performed voltage imaging during the *Pre, Stim*, and *Post* epochs; 27 ± 2 VR trials were recorded for each cell sampling across all three epochs (Figure 2B, n = 32 cells, 8 mice). Optogenetic stimulation targeted to single cells (300 ms duration, 25 mW/mm^2^) readily evoked spikes and plateau potentials that were clearly resolved via holographically targeted voltage imaging (Figures 2B and S2). Remarkably, after optogenetic stimulation, the targeted cells developed spatially modulated firing spanning the location where targeted optogenetic stimulation was delivered (Figures 2B and 2C); the peak in spatially modulated firing was near and slightly prior to the optically stimulated location (Figure 2C). We separately tested optogenetic stimulation at three different virtual-space locations (60, 90, and 120 cm); all induced similar place-field plasticity (Figures 2D and 2E). The peak in spatially modulated firing was reliably backward-shifted relative to the optically stimulated location by −12.9 ± 3.7 cm (Figure 2F, n = 32 cells, p = 0.001, two-sided paired-sample t test), consistent with prior predictive coding results^3^ and models^51^ of the hippocampus. Firing rates 10–30 cm before the optogenetic stimulation location significantly increased in both *Stim* and *Post* epochs compared with the *Pre* epoch (Figure 2F, *Pre:* 1.7 ± 0.4 Hz, *Stim:* 5.5 ± 0.8 Hz, *Post*: 8.6 ± 0.9 Hz, *Pre* vs. *Stim*, p = 4 × 10^−5^, *Pre* vs. *Post,* p=2 × 10^−8^, two-sided paired-sample t test). Cells that did not become classically defined place cells also exhibited significant increases in firing rate near the stimulation-location (Figure S2). Control experiments targeting optogenetic stimulation only to single cells while simultaneously imaging surrounding cells revealed that plasticity was specific to cells receiving optogenetic stimulation (Figures S3A and S3B). To test stability of induced plasticity, we re-imaged the same cells 24 h after optogenetic induction; 6/10 cells were still place cells tuned to the stimulation location (Figures 2G, 2H, S3C, and S3D).

In the optically created place cells (n = 25), in-field firing rate was increased manyfold (Figure 2I, *Post*: 9.0 ± 0.6 Hz; *Pre*: 1.6 ± 0.3 Hz; p = 2 × 10^−10^, two-sided paired-sample t test), but the procedure did not affect out-of-place-field firing rate (Figure 2I, *Post*: 1.6 ± 0.2 Hz; *Pre:* 1.2 ± 0.2 Hz; p = 0.18, two-sided paired-sample t test), indicating that optogenetic plasticity did not simply cause overall increased activity. Optogenetically created place cells exhibited similar in-field and out-of-field firing rates as natural place cells (Figure 2I, in-field: p = 0.66, out-of-field: p = 0.47, two-tailed t test). Optically created place cells also displayed higher in-field complex spikes rate compared with out-of-field (in-field: 0.5 ± 0.06 Hz, out-of-field: 0.04 ± 0.01 Hz, p = 3 × 10^−8^, two-sided paired-sample t test), similar to natural place cells. In animals with experimentally created place cells, we did not observe changes in licking and running behavior (Figure S4). These optogenetic-stimulation results were concordant with prior results on BTSP with intracellular electrical stimulation.^3^

### Subthreshold properties and experimental creation of multiple-cell representations

**Figure 3.**
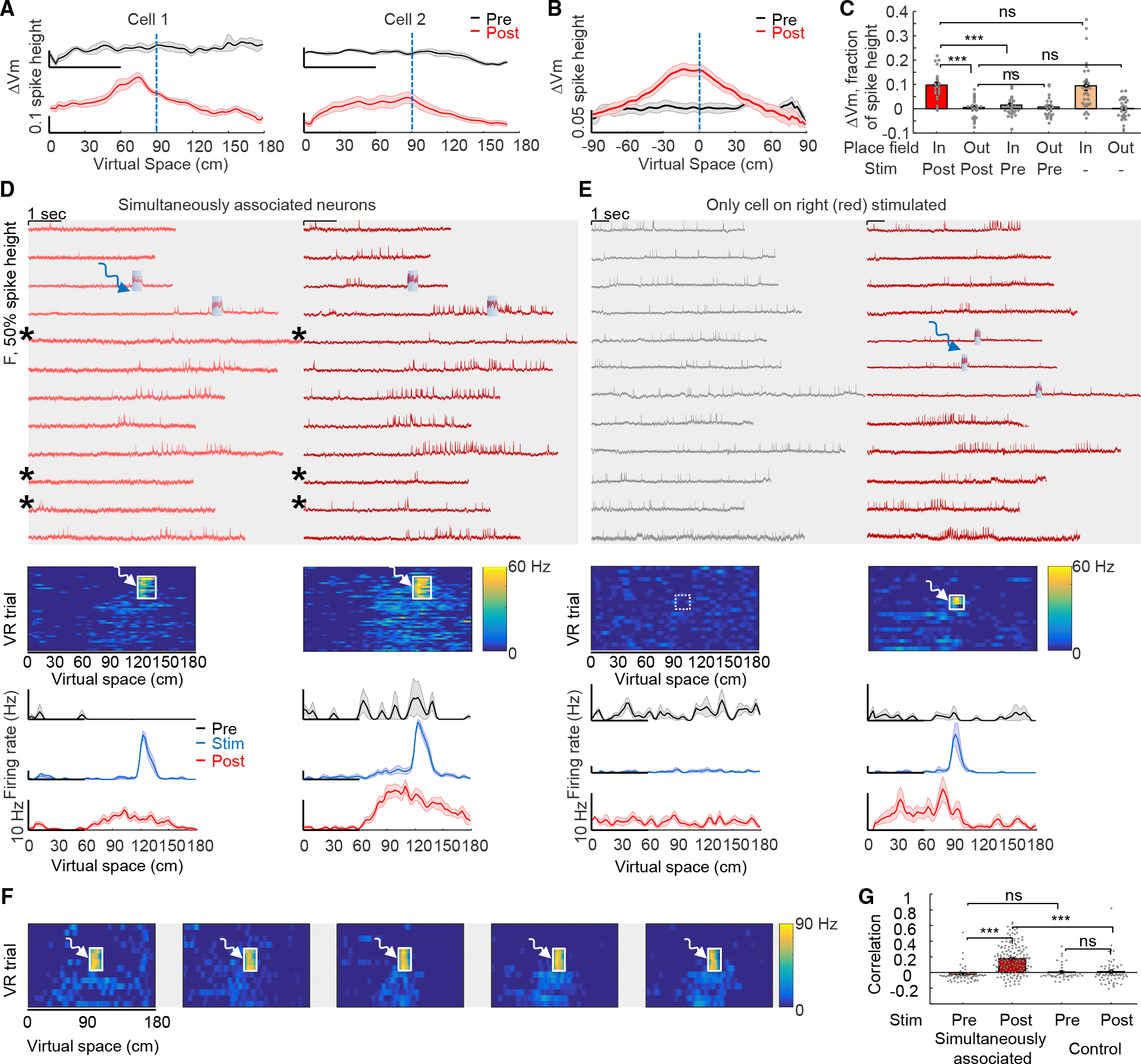
Induced BTSP exhibited enhanced subthreshold potentials and voltage correlations among simultaneously associated neurons (A) Mean subthreshold membrane potential changes (spikes removed, low-pass filtered <3 Hz) for cells shown in Figure 2B. Black: pre-optogenetic stimulation. Red: post-optogenetic stimulation. (B) Mean subthreshold membrane potential (for n = 32 cells aligned at 0 cm). (C) Optogenetic stimulation did not affect out-of-field membrane potential but did increase in-field membrane potential (n = 25 cells). Optogenetically induced place cells had similar relative in-field and out-of-field membrane potential dynamics as natural place cells; values for natural place cells (orange bars, right) from the dataset in Figure 1. (D) Example of simultaneously optically stimulated cells forming stable representations at the same location (n = 18 pairs). Red: GEVI fluorescence traces as a function of time for selected VR trials. Blue: simultaneous 300 ms targeted optogenetic stimulation at 120 cm. Gray shaded zones indicate simultaneously recorded cells. Asterisks indicate concurrent spiking failures inside the place field. Middle: Firing rates from all VR trials (pre-, during, and post-optogenetic stimulation) across virtual space. Bottom: mean firing rate maps. (E) Same as (D) but control condition wherein the left cell is simultaneously imaged but not optogenetically stimulated. (F) Firing rate across virtual space for simultaneously optically stimulated and associated cells (n = 5 cells). (G) Pearson’s correlation coefficients of the firing rate over virtual space between simultaneously stimulated cells and between simultaneously imaged cells under control conditions. All error bars and shading: SEM.

We next sought to identify mechanisms underlying this rapid BTSP, considering first that subthreshold voltage dynamics reflect interaction of synaptic inputs with cellular membrane properties. We observed ramping subthreshold depolarizations—absent during the *Pre* epoch—around and preceding the optogenetic-induction location during the *Post* epoch (Figures 3A and 3B), consistent with the asymmetric plasticity rule.^3^ Subthreshold depolarization 10–30 cm before the optogenetic induction location was significantly higher *Post* compared with *Pre* (Figure 3B, *Pre*: 0.6% ± 1.1% of spike height*, Post*: 8.3% ± 1.3%, n = 32 cells, p = 1.4 × 10^−7^, two-sided paired-sample t test).

For optically created place cells, optogenetic induction did not affect out-of-field subthreshold membrane potential (Figure 3C, post: 0.6% ± 0.8% of spike height, pre: 0.7% ± 0.8%, n = 25 cells, p = 0.88, two-sided paired-sample t test), but significantly enhanced in-field subthreshold potential (Figure 3C, post: 9.7% ± 1.2% of spike height, pre: 1.5% ± 1.0%, p = 4 × 10^−8^, two-sided paired-sample t test). The optogenetically induced place cells displayed naturalistic membrane potential changes (Figure 3C, both in-field and out-of-field values, vs. naturally occurring place cells from Figure 1). Theta-rhythm amplitude was also increased at and near the stimulation location after optogenetic plasticity, resembling properties of naturally occurring place cells (Figure S3). Previously, Lee et al.^48^ showed that electrophysiological depolarization of single CA1 cells could lead to place cell-like spiking; however, generated responses were at locations not specific to the depolarization and immediately disappeared after depolarization, suggesting interaction of unchanged synaptic inputs with transiently altered membrane properties from the manipulation. In contrast, here, the induction of place-field firing and subthreshold membrane potential dynamics were stimulation location-specific and stable, lasting well beyond induction and indeed throughout the epochs following optogenetic stimulation (at least 24 h after induction).

We next considered that intracellular correlations between simultaneously imaged cells could reflect shared inputs. We were able to simultaneously create up to five induced place cells with place fields imposed at the same location (Figures 3D and 3F). On some trials, place cells did not fire action potentials inside the place field as shown earlier (Figure 2B); here, in pairs of simultaneously associated and imaged neurons, we also observed this failed firing in some trials after plasticity induction (Figure 3D, asterisks). Remarkably, spiking failures inside the place fields typically occurred concurrently (Figure 3D, asterisks), suggesting common inputs or shared modulation processes between simultaneously stimulated/imaged cells. Optical place-field induction significantly increased correlation between simultaneously stimulated cells (Figure 3G, pre: −0.02 ± 0.01, post: 0.18 ± 0.01, n = 18 pairs, p = 1 × 10^−8^, two-tailed t test), whereas control experiments (targeting optogenetic stimulation only to single cells while simultaneously imaging surrounding cells) did not change correlations (Figure 3G, pre: 0.09 ± 0.05, post: 0.05 ± 0.04, n = 6 pairs, p = 0.82, two-tailed t test). These results, extending prior single-cell electrophysiology findings to broader local networks, were consistent with a synaptic mechanism for the BTSP.

### No detectable change in excitability with optical place cell creation

**Figure 4.**
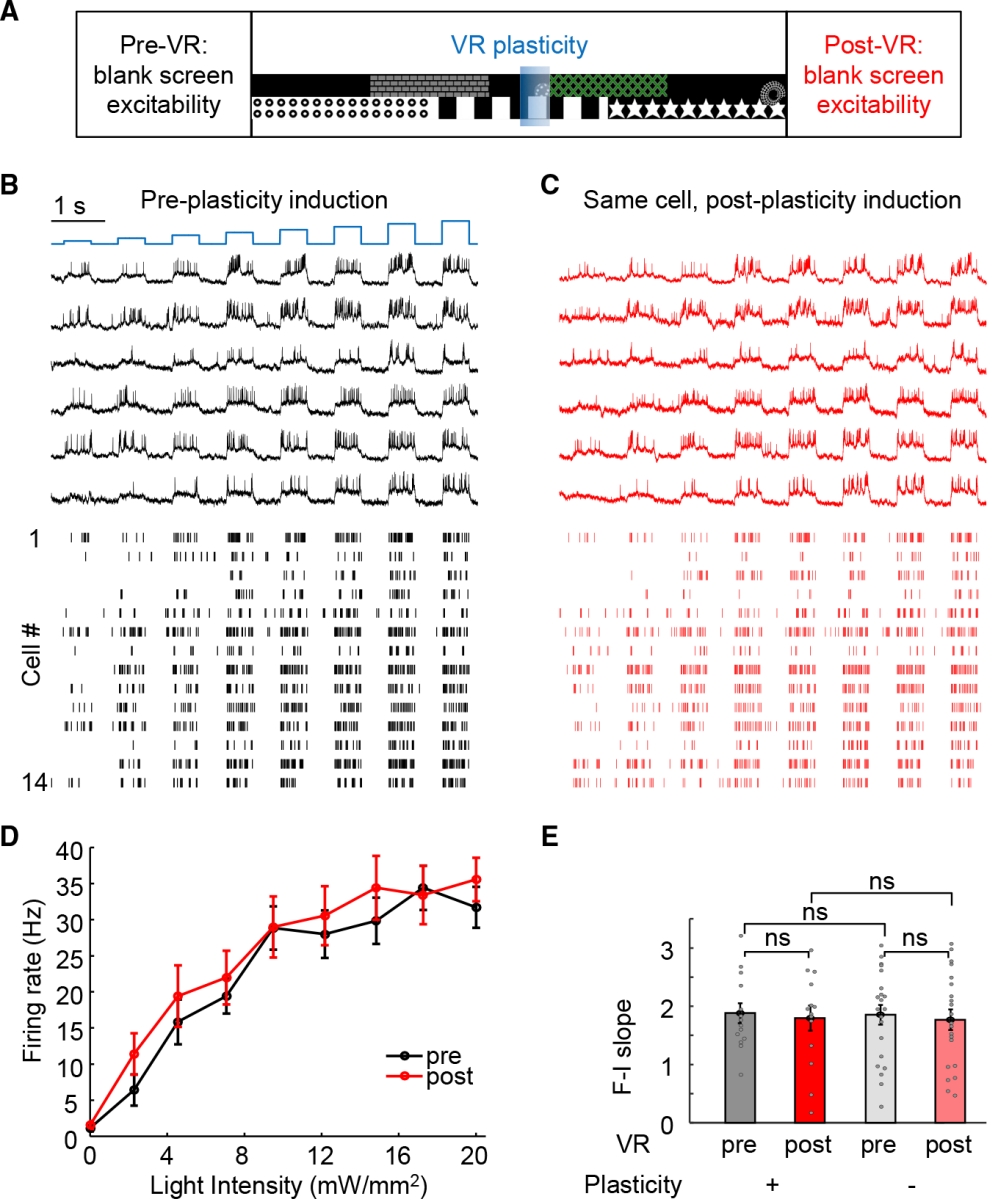
Stability in optically assessed single-cell excitability parameters before vs. after induction of place-field plasticity (A) Excitability was assessed out of the VR environment, before vs. after optogenetically induced plasticity. (B and C) Paired recordings of the same cells before and after optically induced plasticity. Blue: patterned optogenetic stimulation with steps of blue light (500 ms duration, 0–20 mW/mm^2^). Black: example fluorescence of somQuasAr6a during optogenetic stimulation. Red: fluorescence recording of the same cell after optically induced plasticity. Bottom: spike raster before and after optically induced plasticity; n = 14 cells. (D) Mean firing rate as a function of optogenetic stimulus strength (F-I curve) pre- (black) and post-(red) optical plasticity induction. (E) Excitability before and after optical plasticity, summarized for each cell as the slope of the F-I curve from 0–17 mW/mm^2^. Slope of the F-I curve did not change (pre: 1.9 ± 0.2, post: 1.8 ± 0.2, p = 0.67, two-sided paired-sample t test; n = 14 cells; control: pre: 1.9 ± 0.2, post: 1.8 ± 0.2, p = 0.43, two-sided paired-sample t test; n = 21 cells; pre vs. control-pre: p = 0.91, two-tailed t test; post vs. control-post: p = 0.92, two-tailed t test). Error bars/shading: SEM.

The optically induced plasticity did not cause an overall increase in activity of the new place cells (Figure 2I), suggesting no change in excitability. To directly test this important point, we performed all-optical excitability measurements^32,36^ before vs. after optically induced plasticity (Figure 4A). To eliminate confounds from visual events or behavioral states, we performed the excitability measurements in a controlled setting without visual stimuli. In mice running on the spherical treadmill without the VR environment, single-cell-targeted optogenetic stimuli (500 ms/1 Hz, 4.8–20 mW/mm^2^) evoked stimulus intensity-dependent spiking, providing a baseline excitability metric (Figure 4B). The same cells then were carried through the plasticity induction and assessment in the VR environment (as in Figure 2). Finally, the excitability measurements were repeated on the same cells, once again out of the VR environment (Figure 4C). We quantified the relationship between mean firing rate F and optogenetic stimulus strength I (F-I curve) in the same cells before and after optical plasticity (Figure 4D). The spontaneous activity at I = 0 did not change, consistent with results in Figure 2I (pre: 1.2 ± 0.5 Hz, post: 1.6 ± 0.6 Hz, n = 14 cells, p = 0.33, two-sided paired-sample t test). The firing rate at eight different optogenetic excitation-strengths did not change either (p = 0.16, 0.16, 0.41, 0.96, 0.34, 0.12, 0.80, and 0.25; two-sided paired-sample t test). Control experiments without plasticity induction revealed that excitability was stable during the VR behavior (Figures 4E and S5). The slope of the F-I curve did not change pre- vs. post-plasticity (Figure 4E, slope ratio, post/pre: 0.98 ± 0.11, p = 0.67, two-sided paired-sample t test), as with the control conditions without plasticity induction, further confirming that optically induced plasticity in CA1 cells was not due to an excitability increase.

These paired excitability measurements of the same cells before and after plasticity induction addressed whether cells exhibit greater excitability when they became place cells during BTSP but not whether place cells were more excitable than non-place cells. To address this distinct question while eliminating potential confounds from variable opsin expression among cells, we then compared spike-initiation threshold above baseline between place cells (out-of-field) and non-place cells (STAR Methods). This value did not differ (Figure S5; non-place cells: 22.4% ± 0.7% of spike height, n = 71 neurons; place cells outof-field: 21.8% ± 1.5% of spike height, n = 30 neurons, p = 0.69, two-tailed t test). Non-place cells displayed slightly higher firing rate vs. out-of-field firing rate of place cells (Figure S5; non-place cells: 2.5 ± 0.2 Hz, n = 71 neurons; place cells out-of-field: 1.8 ± 0.3 Hz, n = 30 neurons, p = 0.044, two-tailed t test), consistent with prior results.^17^ A previous study^47^ found that place cells exhibited higher excitability comparing with silent cells in anesthetized rats. By contrast, here the excitability profiles were measured in awake animals, and the comparison was not restricted to silent cells; excitability properties are known to be different in anesthetized vs. awake states^36^ and in quiet vs. walking states.^32^

### All-optical interrogation of CA2/3-to-CA1 synapses in behaving mice

**Figure 5.**
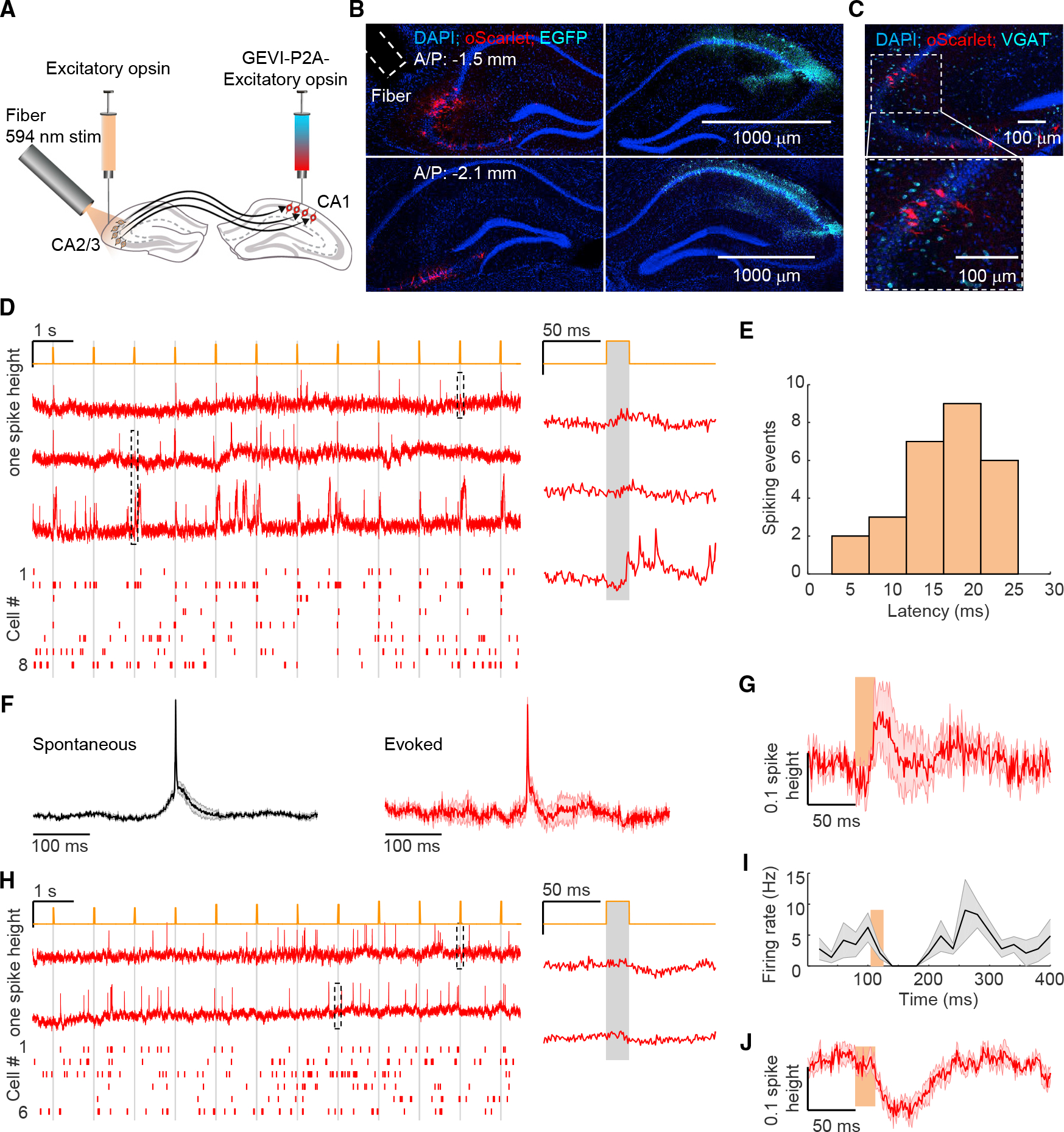
All-optical physiology of CA2/3-to-CA1 synaptic transmission in behaving mice (A) Optical assay of synaptic function between hippocampal CA2/3 and CA1. CAV2-Cre, Flpo and Flp-on-somQuasAr6a(GEVI)-P2A-sombC1C2TG(opsin) were injected into CA1, and Cre-on-ChRmine-oScarlet-Kv2.1 was injected into contralateral CA2/3. (B) Confocal images of fixed brain slices showing fiber positioning and expression of ChRmine (red) in CA2/3 and somQuasAr6a (cyan) in CA1. Scale bars, 1,000 mm (representative data; similar results n = 5 preparations). (C) Representative confocal image of fixed brain slices expressing ChRmine (red) stained with the vesicular GABA transporter (VGAT) (cyan). 103/103 neurons expressing oScarlet were VGAT-negative (n = 5 mice). Bottom: magnified view of the boxed region at top. (D) All-optical assessment of EPSPs. Orange: optical stimulation of CA2/3 neurons through fiber (594 nm, 20 ms duration, repeated at 1 Hz). Left top: example fluorescence traces from CA1 neurons. Bottom: spike raster (n = 8 neurons). Right: expanded fluorescence waveforms from boxed region at left. (E) Distribution of delays between CA2/3 stimulation onset and peak of evoked spike (27 spiking events from 96 trials; latency: 17 ± 6 ms, mean ± SD, n = 8 cells). (F) Spike-triggered average waveform of spontaneous (left) and CA2/3 stimulus-evoked action potentials (right, n = 8 neurons). (G) CA2/3 stimulation-triggered mean fluorescence to stimuli that failed to evoke spikes corresponding to (D). (H) As in (D) except no CA2/3 stimulation-evoked spikes were detected. Instead, decreased fluorescence signal resulted (n = 6 cells). (I) Mean spike rate during CA2/3 stimulation corresponding to (H). (J) CA2/3 stimulation-triggered mean fluorescence corresponding to (H). Error bars/shading: SEM.

Since optogenetically induced plasticity was accompanied by stimulus-location-specific increases in subthreshold membrane potential and theta rhythmicity, as well as concurrency of spiking failures across simultaneously associated neurons (all with no detectable change in excitability), it appeared that optically induced plasticity could arise instead from synaptic plasticity—for example, in the projection from CA3 to CA1^52^ which is critical for both spatial context-dependent responses^53^ and contextual learning.^54^ We therefore developed an all-optical method to probe synaptic transmission between projection-defined genetically targeted presynaptic CA2/3 cells and postsynaptic CA1 cells *in vivo*. We targeted the contralateral CA2/CA3 to ipsilateral CA1 projection (Figure 5A) known to show convergent connectivity with the ipsilateral projection.^55^ This approach uniquely enabled simultaneous optics access to presynaptic and post-synaptic regions while also minimizing optical crosstalk, especially when combined with soma-localized opsin expression—that is, optogenetic stimuli delivered to presynaptic cells could not directly influence postsynaptic cells, and likewise, optical stimulus/imaging light delivered to postsynaptic cells could not exert optical crosstalk upon presynaptic cells. Even dense ChRmine-oScarlet-Kv2.1 expression in CA2/3 revealed undetectable axonal fluorescence in contralateral CA1 (Figure S6), further validating effectively minimized crosstalk from any undesired axonal trafficking of ChRmine from CA2/3 to contralateral CA1.

To target projection-defined presynaptic CA2/3 cells for optical stimulation, we used the retrograde canine adenovirus type 2 (CAV2) encoding Cre recombinase injected into CA1, and Cre-dependent (DIO) ChRmine-oScarlet-Kv2.1 injected into CA2/3 (Figure 5A). We simultaneously expressed somQuasAr6a and sombC1C2TG in postsynaptic CA1 cells (as described above). Confocal imaging of fixed brain slices confirmed that ChRmine expressed sparsely in contralateral CA2/3 cells and was largely restricted to the soma (Figure 5B). *In situ* hybridization for the vesicular GABA transporter (VGAT, an inhibitory marker) confirmed that ChRmine did not express at all in inhibitory neurons (Figure 5C; n = 103 neurons, 5 mice, 9 ± 6 cells per 100 μm slice, mean ± SD).

We implanted a tilted fiber (200 μm diameter, 0.39 numerical aperture (NA), 3 mm long) for optically activating contralateral CA2/3 along with a cannula for imaging CA1 (Figures 5A and 5B; STAR Methods). We first imaged CA1 cells in mice running on the spherical treadmill, with a blank screen while delivering optogenetic stimuli to CA2/3 cells (594 nm, 20 ms duration, ~2.8–6.4 mW/mm^2^, repeated at 1 Hz; STAR Methods) (Figure 5D). Brief optogenetic activation of CA2/3 cells elicited excitatory post-synaptic potentials (EPSPs), and sometimes spiking, in CA1 cells (Figure 5D, 27 spiking events from 96 trials, 8/18 cells, 4 mice). The mean latency from CA2/3 stimulus onset to detected-spike peak (STAR Methods) in CA1 was 17 ± 6 ms (mean ± SD; Figure 5E). STA waveform of spontaneous spikes revealed slowly rising (~50 ms) depolarization that preceded the action potential, whereas CA2/3 stimulus-evoked STA arose more swiftly, consistent with precisely timed and synchronized CA2/3 stimulus-evoked synaptic excitation onto CA1 neurons (Figure 5F). CA2/3 stimulus-triggered average waveform of trials that did not induce spikes revealed the underlying evoked EPSP (Figure 5G, 12.4% ± 5.5% of spike height, n = 8 cells). Together, these properties were consistent with optically triggered monosynaptic transmission from CA2/3 to CA1,^40,56^ although longer-latency contributions from polysynaptic transmission may also certainly occur.

In a subset of cells (n = 6/18), CA2/3 test-pulses induced significant decreases in CA1 spike rate (Figures 5H and 5I; detected 25–85 ms after stimulus onset, p = 0.04, two-sided paired-sample t test) and indeed evoked a robust hyperpolarization (Figure 5J, −15.5% ± 1.8% of spike height), consistent with elicitation by an IPSP. We then compared the relative timing of this IPSP and the more common EPSPs; from stimulus onset, the latency of depolarization onset was ~12 ms, and latency of hyperpolarization onset was ~16 ms. This short delay between onset of excitation and inhibition was consistent with well-known feedforward synaptic inhibition mechanisms in the hippocampus.^57,58^ Together, these results established all-optical quantitative measurement of synaptic transmission between projection-defined CA2/3 and individually defined CA1 cells in behaving mice.

### Potentiation of synaptic transmission in optogenetically induced place field plasticity

**Figure 6.**
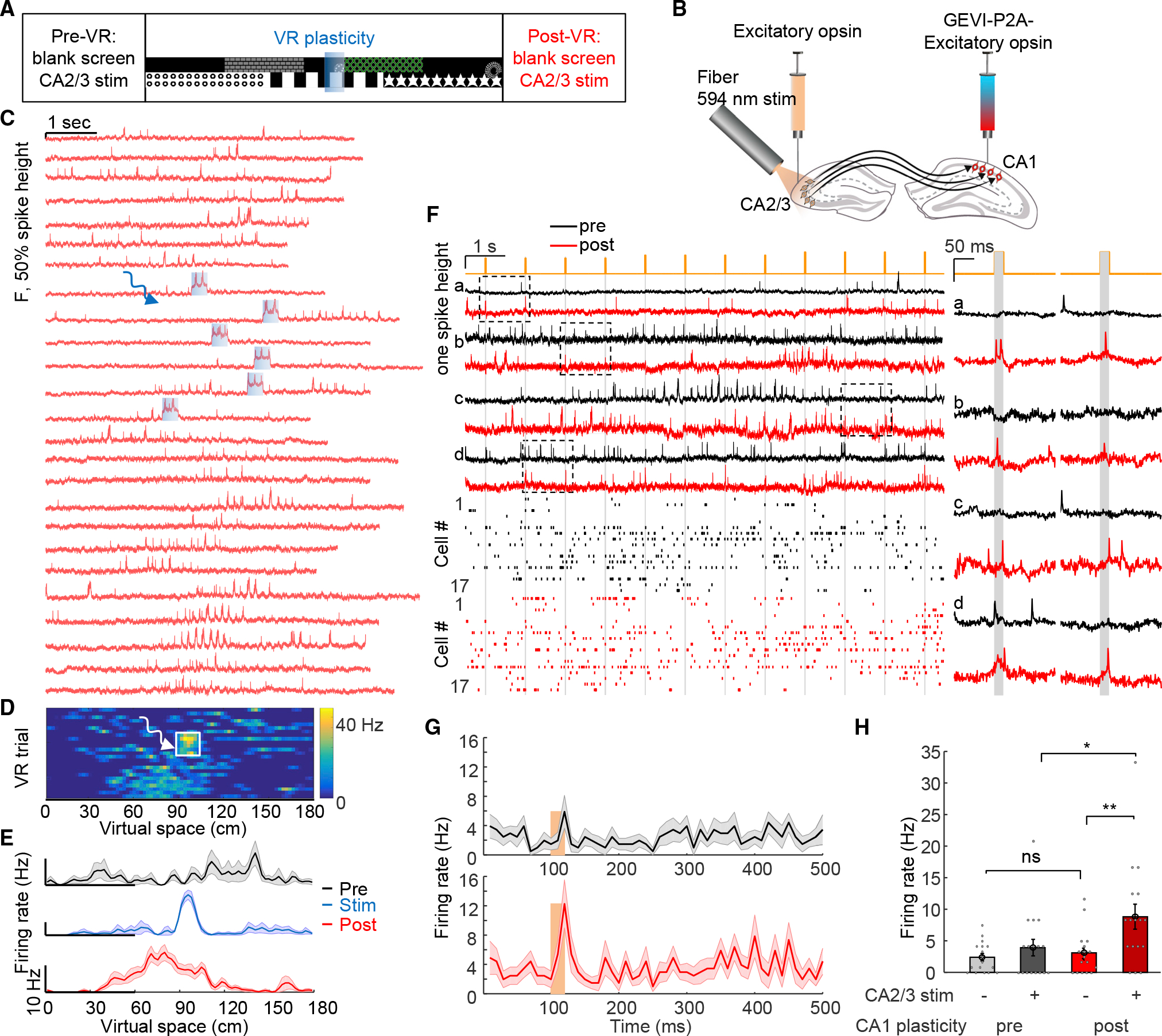
Potentiation of CA2/3-to-CA1 synaptic inputs by optogenetically induced plasticity (A and B) Synaptic function assessed out of VR environment before vs. after optogenetically induced plasticity. (C–E) Example of CA1 plasticity induction by optogenetic stimulation of CA1 cells as in Figure 2, but in animals configured as in (B) with soma-localized ChRmine expression and fiber implantation in CA2/3. (F) Top: examples of paired measurements of fluorescence signals from the same cells in response to CA2/3 stimulation, pre- vs. post- CA1-induced plasticity. Bottom: corresponding spike raster, pre- vs. post-CA1-induced plasticity. Right: magnified views of the boxed regions at left. (G) Mean spike rate during CA2/3 stimulation pre- and post-opto-plasticity. (H) Quantification of CA2/3 stimulation effect, pre- vs. post-CA1-induced plasticity. CA2/3 stimulation evoked a substantial increase in spike rate post CA1-induced plasticity, measured over 1–20 ms window following CA2/3 test-pulse onset (n = 17 cells). Importantly, the spontaneous firing rate defined by the 200 ms time window before the CA2/3 test-pulses did not change. Error bars/shading: SEM.

We next sought to probe the modulation of synaptic transmission between CA2/3 and CA1 before vs. after optogenetically induced place field plasticity (Figures 6A and 6B; STAR Methods). In mice running on the spherical treadmill out of the VR environment, we imaged CA1 cells while delivering brief optogenetic test-pulses to contralateral CA2/3 cells (594 nm, 20 ms duration, 1 Hz) to measure synaptic effects (Figure 6A, black). The same CA1 cells were then stimulated for plasticity induction and assessed in the VR environment (as in Figure 2). Finally, optogenetic test-pulses were delivered again to the same contralateral CA2/3 cells while the same CA1 cells were imaged, once again out of the VR environment (Figure 6A, red). To assess the stability of synaptic transmission during the VR behavior, we included a control condition replicating the structure of the experiment but without plasticity induction in CA1 (Figure S6).

In the configuration with soma-localized ChRmine expression and fiber implantation in CA2/3, optogenetic stimulation of CA1 cells induced BTSP as before (Figures 6C–6E, as in Figure 2). We then compared CA2/3 test-pulse-induced responses in the same CA1 cells before vs. after optically induced BTSP (Figure 6F). Some CA1 cells that showed EPSPs (Figure 6F, e.g., cell a) or IPSPs (Figure 6F, e.g., cell c) in response to CA2/3 test-pulses before the plasticity induction showed reliable spiking in response to the same CA2/3 test-pulses after plasticity induction. CA1 cell-targeted plasticity induction significantly increased CA2/3 stimulus-induced spike rates in CA1 cells, measured over the window 1–20 ms following CA2/3 stimulus onset (Figures 6G and 6H, pre-plasticity: 3.9 ± 1.3 Hz vs. post-plasticity: 8.8 ± 2.0 Hz, n = 17 cells, 5 mice, p = 0.02, two-sided paired-sample t test). Spontaneous spike rates, measured over the 200 ms time window before CA2/3 test-pulses to avoid confounds from test-pulse effects, did not change (pre-plasticity: 2.4 ± 0.6 Hz, post-plasticity: 3.1 ± 0.8 Hz induction, p = 0.30, two-sided paired-sample t test). Control experiments without plasticity induction revealed that synaptic transmission was stable during VR behavior alone (Figure S6). Together, these results demonstrated potentiation of CA2/3-to-CA1 synaptic inputs onto the stimulated CA1 cells during BTSP in behaving mice.

### Presynaptic CA2/3 activity required for plasticity induction in CA1

**Figure 7.**
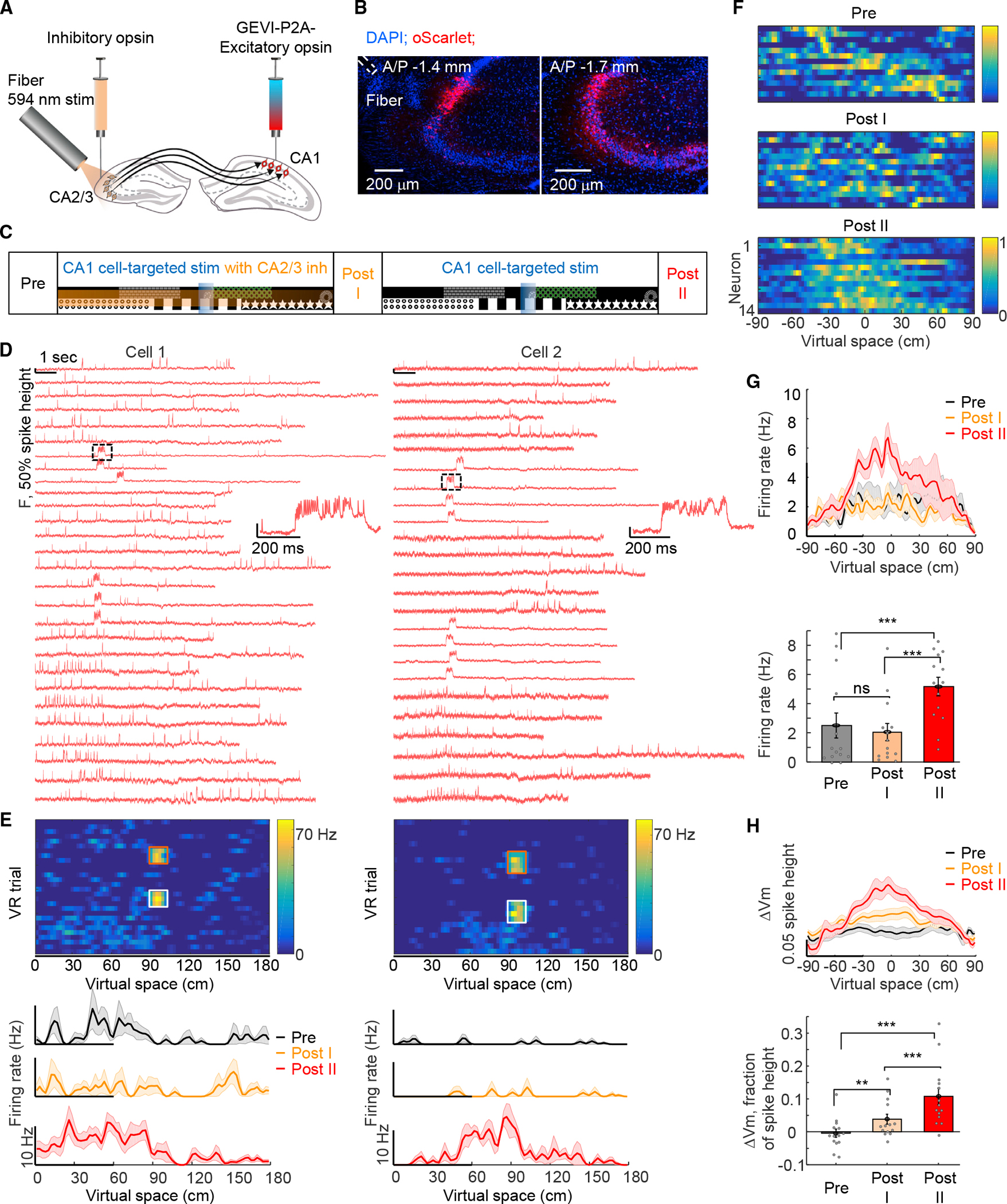
Optogenetic silencing of CA2/3 reveals role of CA2/3 activity for full plasticity induction in CA1 (A) Optogenetic inhibition of CA2/3 during CA1-targeted optogenetic stimulation. Flpo and Flp-on-somQuasAr6a(GEVI)-P2A-sombC1C2TG(excitatory opsin) were injected into CA1, and eHcKCR1–3.0(inhibitory opsin)-oScarlet-Kv2.1 into contralateral CA2/3. (B) Confocal images of fixed brain slices showing fiber positioning and expression of eHcKCR1–3.0 (red) in CA2/3. Scale bars, 200 μm (representative data; similar results n = 3 preparations). (C) Closed-loop CA1-targeted optogenetic stimulation at specific locations with or without optogenetic silencing of CA2/3 neurons. (D) Two example cells: fluorescence traces as a function of time for VR trials during *Pre, Stim* with CA2/3 inhibition, *Post I, Stim* without CA2/3 inhibition, and *Post II* epochs. Insets on the right: magnified views of the black dashed regions at left. (E) Firing rate of all VR trials (*Pre, Stim* with CA2/3 inhibition, *Post I, Stim* without CA2/3 inhibition, and *Post II* epochs) across virtual space for the two cells shown in (D). Orange and white boxes: 300 ms targeted optogenetic stimulation at 90 cm location with (orange box) and without (white box) CA2/3 inhibition. Bottom: average firing rate maps for the cells shown in (D). Black: *Pre*. Orange: *Post I*. Red: *Post II.* (F) Normalized firing rates of all cells (n = 14 cells). Optogenetic stimulation locations were at aligned at 0 cm. (G) Top: average firing rate maps of n = 14 cells aligned at 0 cm. Bottom: quantification of firing rate at 10–30 cm before the optically stimulated location for *Pre, Post I*, and *Post II* epochs. (H) Top: mean subthreshold membrane potential of n = 14 cells aligned at 0 cm. Bottom: subthreshold membrane potential at 10–30 cm before the optically stimulated location. Subthreshold potential was significantly higher in both *Post I* and *Post II* epochs compared with the *Pre* epoch. Error bars/shading: SEM.

These results demonstrated potentiation of CA2/3-to-CA1 synaptic transmission by optogenetically induced BTSP but did not prove that presynaptic CA2/3 activity was required for the observed plasticity. To address this question (Figure 7A), we generated an especially potent light-gated potassium channel HcKCR1^59^ with three trafficking sequences (eHcKCR1–3.0; STAR Methods). Confocal imaging of fixed brain slices confirmed that eHcKCR1–3.0 was largely restricted to the soma in contralateral CA2/3 cells (Figure 7B). Whole-cell recordings in acute slices expressing eHcKCR1–3.0 confirmed that optogenetic stimuli efficiently inhibited action potentials (Figure S7). We implanted a tilted fiber (200 μm diameter, 0.39 NA, 3 mm long) for optically inhibiting contralateral CA2/3 along with a cannula for imaging in CA1 (Figures 7A and 7B; STAR Methods).

We performed closed-loop CA1-targeted optogenetic stimulation at specific locations during the VR behavior, with or without optogenetic silencing of CA2/3 cells, with two blocks in one behavioral session. For the first block, the mouse ran for 20 trials (*Pre* epoch, Figure 7C), whereupon 300 ms CA1-targeted optogenetic stimulation was delivered at the 90 cm location for 10 trials, whereas CA2/3 inhibition was simultaneously delivered across the whole virtual space for 10 trials (*Stim with CA2/3* inh epoch, Figure 7C; inhibition light: 594 nm, ~2.8–6.4 mW/mm^2^; STAR Methods), and then, the animal ran for another 30 trials with neither CA1 nor CA2/3 stimulation (Post I epoch, Figure 7C). The second block included the 300 ms CA1-targeted optogenetic stimulation at the 90 cm location for 10 trials without CA2/3 inhibition (*CA1 cell-targetedStim* epoch, Figure 7C), and finally, the animal ran for another 30–50 trials (*Post II* epoch, Figure 7C). To eliminate confounds of gradual development of plasticity, we included a control session replicating the temporal structure of the experiment but lacking CA2/3 inhibition in the first block (Figure S7).

CA1-targeted optogenetic stimulation, in the absence of optogenetic inhibition of CA2/3, induced spatially modulated firing plasticity in the second block (Figures 7D–7G; firing rates 10–30 cm before the optogenetic stimulation location, *Post I:* 2.1 ± 0.6 Hz, *Post II:* 5.2 ± 0.6 Hz, n = 14 cells, 2 mice, p = 5 3 10^-4^, two-sided paired-sample t test), as in earlier experiments (Figure 2). Remarkably, the same cells recorded in the same behavior session did not develop spiking plasticity in response to CA1-targeted optogenetic stimulation in the presence of optogenetic inhibition of CA2/3 (Figures 7D–7G, *Pre:* 2.5 ± 0.9 Hz, *Post* I: 2.1 ± 0.6 Hz, p = 0.59, two-sided paired-sample t test). Notably, CA1-targeted optogenetic stimulation in the presence of optogenetic inhibition of CA2/3 readily evoked plateau potentials as in previous experiments (Figure 7D).

The subthreshold depolarization in the *Post II* epoch was significantly higher compared with that of the *Pre* epoch (Figure 7H, *Pre*: −0.4% ± 1.3% of spike height, *Post II*: 10.8% ± 2.5% of spike height, p = 1.8 × 10^−4^, two-sided paired-sample t test), closely matching the asymmetric plasticity results in Figure 3. The same cells in the *Post I* epoch showed much lower subthreshold depolarization compared with the *Post II* epoch but significantly higher subthreshold depolarization compared with the *Pre* epoch, suggesting a partial inhibition of plasticity by contralateral CA2/3 silencing (Figure 7H, *Post I*: 3.9% ± 1.5% of spike height, *Post I* vs. *Post II*, p = 0.003, *Post* I vs. *Pre*, p = 0.02, two-sided paired-sample t test). We speculate that this partial subthreshold potentiation arises from uninhibited ipsilateral CA2/3 synapses or entorhinal synapses.^60^ Control experiments without CA2/3 inhibition but with the same temporal structure revealed that CA1-targeted optogenetic stimulation in the first block immediately induced full plasticity both for spiking and subthreshold dynamics (Figure S7). Together, these results establish that optically induced BTSP in CA1 is dependent on presynaptic CA2/3 activity, consistent with the model^3^ that a subset of CA2/3 inputs active nearby the stimulus-location in CA1 are potentiated.

## DISCUSSION

Here, with optogenetic tool development (STAR Methods) alongside improved GEVI technology,^41^ we achieved high-sensitivity genetically targeted recording and manipulation of membrane voltage in the hippocampus of mice performing VR spatial behavior. In the course of this work, we were able to identify critical features and mechanistic underpinnings of behavioral timescale plasticity in the brains of living mice. The approach described here provides a potentially generalizable all-optical framework for studying the natural and causal cell-specific processes of plasticity in the neural circuits and synapses of behaving mammals.

### Testing for effects on neuronal excitability during plasticity

Here, we found that genetically targeted voltage imaging enabled quantification of both suprathreshold and subthreshold signatures of physiological voltage dynamics, with single-cell resolution, during behavioral timescale plasticity. By combining genetically targeted voltage imaging and patterned optogenetic activation, we induced and recorded hippocampal BTSP all-optically, finding that optogenetically induced plasticity in place field formation could persist for at least 24 h and could be extended to multiple targeted neurons (moving beyond single-cell intracellular recordings of prior results). Although neuronal excitability changes would have been a plausible mechanism for these effects, our all-optical measurements of excitability before vs. after rapid place field formation revealed no changes in excitability.

Of note, we also did not find evidence that naturally occurring place cells exhibit higher excitability than non-place cells. Although cells do not appear to become more excitable when developing these place cell properties, other forms of plasticity such as homeostatic plasticity^61^ could still be present. The ability to study excitability mechanisms^32,36^ with single-cell resolution, across many cells *in vivo*, opens up the possibility of examining—beyond place cell formation—the role of excitability modifications in other types of learning and memory^62^ in behaving animals.

### Synaptic mechanisms underlying plasticity

Further addressing mechanism, we developed optogenetic tools to measure and manipulate synaptic transmission between projection-defined genetically targeted presynaptic and postsynaptic neurons in behaving mice. We found that BTSP was accompanied by stable changes in dynamics of subthreshold membrane potential and plateau-driven complex spikes and by increased correlation among simultaneously associated neurons, consistent with a synaptic mechanism for the plasticity. We further demonstrated that projection-defined genetically targeted presynaptic CA2/3 to CA1 synaptic transmission was potentiated in BTSP and that presynaptic CA2/3 activity was required for full induction of BTSP in CA1. Prior, all-optical approaches have combined Ca^2+^ imaging with either single-cell-targeted optogenetics^17,31^ or single-cell-targeted electroporation of opsins^29,30^; of note, these early studies did not give a consistent picture of rapid location-specific place field formation (in all of this work, optical crosstalk from imaging^63,64^ has the potential to induce non-location-specific place field responses^48^), and it was unclear if plateau potentials had been induced.

Here, place field responses were location specific (although we noted a clear backward shift in the induced place field relative to the site of plasticity induction). Given our evidence for synaptic implementation of hippocampal BTSP, and considering the significant backward shift observed in the induced place field, we speculate that the timing of relevant activity in CA2/3^65,66^ may be important for BTSP. Thus, a clear goal for future work will be to resolve and manipulate presynaptic CA2/3 cellular activity with high spatiotemporal resolution, to thoroughly explore the distinctions between BTSP and Hebbian plasticity, and to manipulate synaptic plasticity to further refine the causal link between synaptic plasticity and learning.

### Addressing the complexity of mammalian CNS microcircuitry

Combining genetically encoded voltage imaging with optogenetic stimulation and inhibition, as defined here, enables investigation of well-defined cellular-resolution synaptic and circuit dynamics during behavior, which may be extended to other synapses and circuits. Although previous studies^53^ have shown that ipsilateral CA3 inputs contribute to place field responses, our results demonstrate that contralateral CA2/3 inputs clearly also contribute to CA1 place cell formation (consistent with the known convergence of ipsilateral and contralateral projections^55^), and it will be interesting to directly compare relative contributions of the two pathways. Recent work^67^ has indicated that entorhinal cortical inputs are required for BTSP in CA1. Indeed, our results complement the model that entorhinal cortex drives plateau potentials that may in turn potentiate effects of CA2/3 synaptic inputs during BTSP.

Considering the role of place cells in memory-guided spatial behavior,^17^ in the future, it may be interesting to induce reward-associated place cells to study how newly formed place cells integrate into existing and downstream networks and influence memory-guided spatial behavior.^30^ Directly observing the integration of newly formed place cells into existing networks will require voltage imaging of a larger population of cells, which will be feasible with further development of the genetically encoded voltage sensors, optogenetic actuators/inhibitors, and high-speed optical systems. This work provides a roadmap for such future integration of all-optical fast physiology with sophisticated behavioral paradigms to achieve detailed mechanistic insights into the circuit and synaptic mechanisms underlying cognition, learning, and memory.

### Limitations of the study

Although projection-defined and genetically targeted CA2/3-to-CA1 synaptic transmission was resolved here to study the synaptic potentiation underlying BTSP, we do not rule out potentiation of other synapses onto CA1 neurons (for instance, the synapses from entorhinal cortex^60^) or additional polysynaptic or circuit effects. Future experiments using the optical tools developed here may be needed to address excitatory synaptic dynamics from the entorhinal cortex^67^ to CA1 in plasticity over different timescales. Local inhibitory synapses may also be plastic^68,69^ during BTSP, however, reorganization of local inhibition has been suggested to generate place fields in locations not specific to stimulation sites,^70^ unlike the process investigated here. Furthermore, a recent study^29^ found that local inhibitory neuron activity was constant during and immediately after place field emergence. However, such place cell emergence in fact may trigger downstream local inhibitory circuit changes after home-cage rest periods.^29^ Because the genetically encoded voltage sensor described here is compatible with optogenetic stimulation suitable for optically resolving IPSPs,^36^ this approach may allow for comparably detailed study of inhibitory synaptic plasticity in the course of future exploration.

## STAR★METHODS

### RESOURCE AVAILABILITY

#### Lead contact

Further information and requests for resources and reagents should be directed to and will be fulfilled by the lead contact, Karl Deisseroth (deissero@stanford.edu).

#### Materials availability

Plasmids generated in this study and their sequences are available from Addgene.

Voltage imaging data is available on the DANDI archive (DANDI: 000399, https://dandiarchive.org/dandiset/000399). Other materials used in the analysis are freely available upon request.

#### Data and code availability

- Voltage imaging data is available on the DANDI archive (DANDI: 000399).
- Other materials used in the analysis are freely available upon request.
- Any additional information required to reanalyze the data reported in this paper is available from the lead contact (KD) upon request.

### EXPERIMENTAL MODEL AND SUBJECT DETAILS

#### Primary neurons

All procedures involving animals were in accordance with the National Institutes of Health Guide for the care and use of laboratory animals and were approved by the Institutional Animal Care and Use Committee at Stanford University.

Hippocampal neurons from postnatal day 0 (P0) rat pups (Charles River) were dissected and cultured as previously described.^73^ In brief, P0 neurons were plated in Neurobasal-A medium (Invitrogen) containing 1.25% FBS (Fisher Scientific), 4% B-27 supplement (Gibco), 2 mM Glutamax (Gibco) and 2 mg/mL fluorodeoxyuridine (FUDR, Sigma) on 12 mm glass coverslips pre-coated with 1:30 Matrigel (Beckton Dickinson Labware) in a 24-well plate at a density of ~65,000 cells per well. Neurons were transfected between 6 days and 10 days in vitro using the calcium phosphate transfection method. Measurements on neurons were taken between 4 – 6 days after transfection.

#### Mice

All procedures involving animals were in accordance with the National Institutes of Health Guide for the care and use of laboratory animals and were approved by the Institutional Animal Care and Use Committee at Stanford University.

For *in vivo* experiments, 8–12-week-old wild-type C57BL/6 mice were used. All mice were housed on reverse 12-hour light/dark cycles, with food ad *libitum* and water (outside of behavior training). During training, mice were water-restricted to reach ~80–85% of their initial body weight. Mice of both sexes were used.

### METHOD DETAILS

#### Design of the somQuasAr6a/sombC1C2TG pair

bC1C2TG is a point-mutated chimera with segments from channelrhodopsin-1 (ChR1, amino acids 51–238) and channelrhodopsin-2 (ChR2, amino acids 1–11 and 200–309) guided by our prior work,^43,45^ and mutation at T159G for blue-shifting the action spectrum as in Kato et al.^74^. The final construct (somQuasAr6a-EGFP-P2A-sombC1C2TG) was cloned into an AAV vector with Flp-dependent expression driven by the hSyn promoter. High-titer AAV2/9 virus with somQuasAr6α-EGFP-P2A-sombC1C2TG (1.46×10^13^ GC/mL, LZF2039CR) was obtained from the Janelia Farm Vector Core. High-titer AAV8 virus with CaMKIIα-Flpo (1.69×10^13^ GC/mL) was obtained from the Stanford Gene Vector and Virus Core. High-titer CAV2 virus with Cre (1.25×10^13^ GC/mL) was obtained from the Core CNRS Vector Core. High-titer AAV8 virus with CaMKIIα-DIO-ChRmine-oScarlet-Kv2.1 (4.96×10^12^ GC/mL) was obtained from the Stanford Gene Vector and Virus Core. High-titer AAV9 virus with CKII(0.4)-Cre (2.4×10^13^ GC/mL) was obtained from Addgene. High-titer AAV8 virus with EF1α-DIO-oScarlet (2.50×10^12^ GC/mL) was obtained from the Stanford Gene Vector and Virus Core.

pAAV_CaMKIIa-eHcKCR1–3.0-oScarlet-Kv2.1 was cloned into an AAV vector with the CaMKIIa promoter by inserting the human codon optimized gene of KCR1^59^ fused to oScarlet and Kv2.1. The trafficking sequence (TS) and endoplasmic reticulum export element (ER) were also added to improve the membrane trafficking.^75^ High-titer AAV8 virus with CaMKIIα-eHcKCR1–3.0-oScarlet-Kv2.1 (1.2×10^13^ GC/mL) was obtained from the Stanford Gene Vector and Virus Core.

#### Optical system

The optical system combined a voltage imaging microscope^36^ with a two-photon microscope (Bruker, Ultima). Voltage imaging paths were guided into a Bruker two-photon microscope body via a folding mirror, along with a short-pass dichroic (AVR optics, FF880-SDI01-T3–25X38) to combine with the two-photon beams. Several modifications were made to the voltage imaging microscope.

##### Red laser path

A red laser (CNI Inc., MLL-FN-639, λ = 639 nm, 1000 mW single transverse mode) was attenuated with a half-wave plate and polarizing beam splitter, expanded to a collimated beam of ~42 mm diameter, then projected onto the surface of a reflection-mode liquid crystal spatial light modulator (BNS) as with the macroSLM^73^ with a resolution of 1536×1536 pixels. Polarization of the beam was set with a zero-order half-wave plate. Zero-order diffraction was blocked by a custom anti-pinhole comprised of two magnetic beads (K&J Magnetics, D101-N52) on each side of a glass slide (VWR, Menzel Glaser, 630–2129), placed in a plane conjugate to the sample image plane. The SLM was re-imaged onto the back-focal plane of the objective via a series of relay optics and the Bruker two-photon microscope. Objective lenses were a 25× water immersion objective with numerical aperture 1.05 (Olympus, XLPLN25XWMP2), and a 25× water immersion objective with numerical aperture 1.00 (Leica, HC IRAPO L 25x/1.00 W motCORR). A mechanical shutter blocked the red laser between data acquisitions and a series of OD filters were placed after the red laser for modulating intensity.

The SLM device was controlled by custom software. A user specified area for the SLM to target by drawing on a wide-field epifluorescence image or a 2P fluorescence image. To reduce motion artifact, illumination targeted to somas were used in majority experiments where motion artifacts were observed. The SLM phased pattern was calculated using the Gerchberg-Saxton algorithm. Red laser intensity was ~ 2.5 – 3 mW per cell for *in vivo* imaging.

##### Blue laser path

A blue laser (Coherent, Obis series, λ = 488 nm, 150 mW) was expanded to a collimated beam of ~17 mm diameter, then projected onto a digital micromirror device with a resolution of 1024 × 768 pixels (DMD, Vialux, V-7001 VIS). The patterned blue beam was combined with the patterned red beam via a dichroic mirror. The DMD was re-imaged onto the sample at a magnification such that one DMD pixel corresponded to 0.88 mm in the sample plane. The DMD optical system enabled patterned blue light stimulation across a field of view of ~670 x ~890 μm.

##### Wide-field fluorescence imaging path

The image was relayed from the objective to the camera via a series of three lenses including the tube lens inside the commercial Bruker two-photon microscope. Fluorescence was collected on a scientific CMOS camera (Hamamatsu ORCA-Fusion). The final magnification of the optical system was 12.5, corresponding to 0.52 μm in the sample plane per camera pixel. Fluorescence from the sample was separated from the blue and red excitation beams via a dichroic mirror (Di03-R405/488/561/635-t3–40×55). An emission filter (Semrock 635 nm long-pass, BLP01–635R-25) further separated somQuasAr6a fluorescence from scattered excitation light. An IR-blocking emission filter (Semrock, BSP01–785R-25) was placed for blocking scattered infrared excitation light. All movies are acquired at 1 kHz. To image at 1 kHz, the camera region of interest (ROI) was restricted to typically 200 rows, centered on the image-sensor midline.

##### Contralateral fiber stimulation path

For optogenetic modulation of CA2/3, a 594 nm laser (Hubner, Cobolt Mambo series, λ = 594 nm, 100 mW) was focused into a multi-mode optical fiber (Thorlabs, RJPSL4) and coupled into a 200 μm core diameter, 0.39 NA, 3 mm optical fibers (Thorlabs, FT200EMT) implanted over CA2/3. For optogenetic activation and inhibition, 1.4 – 3.2 mW at the 200 mm fiber cannula tip was used. This corresponds to 45 – 102 mW/mm^2^ at the 200 μm fiber cannula tip, and ~2.8 – 6.4 mW/mm^2^ at the 400 μm depth around the target cells.^76^

##### Control software

The entire setup was controlled by custom software written in LabView. Interfacing was via a National Instruments DAQ (NI cDAQ-9178). The software contained routines for registration of the DMD, SLM, 2P microscope coordinates to the camera via affine transformations.

#### Electrophysiology in primary neurons

##### Optics for electrophysiology in primary neurons

A red laser (CNI Inc., MRL-FN-639, λ = 639 nm, 800 mW single transverse mode) was attenuated with a half-wave plate and polarizing beam splitter, coupled into the Olympus BX61WI microscope via an optical fiber. Blue light was delivered with the Spectra X Light engine (Lumencor) connected to the microscope.

Electrophysiology experiments were performed in extracellular tyrode medium containing (in mM): 150 NaCl, 4 KCl, 2 CaCl_2_, 2 MgCl_2_, 10 HEPES, 10 glucose (pH 7.4). Primary neurons were supplemented with tetrodotoxin (TTX, 1 μM, Tocris) to prevent recruitment of voltage-gated sodium channels. Borosilicate patch pipettes (Harvard Apparatus) were pulled to a tip resistance of 4–6 MΩ, and filled with internal solution containing (in mM):140 potassium gluconate, 10 EGTA, 2 MgCl_2_, 10 HEPES (pH 7.2). Voltage-clamp recordings were acquired using a Multiclamp 700B amplifier (Molecular Devices), filtered at 1 kHz with the internal Gaussian filter and digitized with a DigiData 1440A (Molecular Devices) at 20 kHz. Cells were held at resting potential of −65 mV.

#### Electrophysiology in acute slices

Recordings of pyramidal cells expressing eHcKCR1–3.0 were performed in acute slices from wild-type C57BL/6 mice 4–5 weeks after virus injection. Coronal slices 300 μm in thickness were prepared after intracardial perfusion with ice-cold N-methyl-d-glucamine (NMDG) containing cutting solution (in mM): 93 NMDG, 2.5 KCl, 25 glucose, 1.2 NaH_2_PO_4_, 10 MgSO_4_, 0.5 CaCl_2_, 30 NaHCO_3_, 5 Na-ascorbate, 3 Na-pyruvate, 2 thiourea, and 20 HEPES (pH 7.3–7.4). Slices were incubated for 12 min at 34 °C, and then were transported to room temperature oxygenated artificial cerebrospinal fluid (ACSF) solution containing (in mM): 124 NaCl, 2.5 KCl, 24 NaHCO_3_, 2 CaCl_2_, 2 MgSO_4_, 1.2 NaH_2_PO_4_, 12.5 glucose, and 5 HEPES (pH 7.3–7.4).

Borosilicate patch pipettes (Harvard Apparatus) with resistance of 4–6 MΩ were filled with intracellular medium containing (in mM): 130 potassium gluconate, 20 KCl, 0.2 EGTA, 4 Na_2_ATP, 0.3 NaGTP, 5 Na_2_-Phosphocreatine, 1 MgCl_2_, and 10 HEPES (pH 7.2). Green light was delivered with the Spectra X Light engine (Lumencor) connected to the fluorescence port of a Olympus BX61WI microscope with a 513 nm filter. Current-clamp recordings were acquired using a Multiclamp 700B amplifier (Molecular Devices), filtered at 1 kHz with the internal Gaussian filter and digitized with a DigiData 1440A (Molecular Devices) at 20 kHz. Current injections were delivered at 10 Hz at 1 ms pulse widths past the rheobase of the patched neuron (generally 300–700 pA). To assess inhibition, 513 nm light at 1.0 mW/mm^2^ intensity was delivered continuously for 2 s during pulsed current injections.

#### Cranial windows, virus injections and fiber implantation

##### CA1 virus injection

Virus injections were made using home-pulled micropipettes (Sutter P1000 pipette puller), mounted in a microinjection pump (World Precision Instruments Nanoliter 2010) controlled by a microsyringe pump controller (World Precision Instruments Micro4). The micropipette was positioned using a stereotaxic instrument (Kopf Instruments). For experiments only accessing CA1, virus comprised AAV2/9 hSyn-fDIO-somQuasAr6a-EGFP-P2A-sombC1C2 (final concentration ~2×10^12^ GC/mL) mixed with AAV2/8 CaMKIIα-Flpo (final concentration ~0.5 – 1×10^10^ GC/mL). Virus were injected in the right hippocampal CA1 (250 – 300 nl, 45 – 60 nL/min, AP: −2.1 mm, ML: 1.9 mm, DV: −1.45 mm).

##### CA2/3 virus injection

For experiments activating CA2/3, the virus for CA1 injection comprised AAV2/9 hSyn-fDIO-somQuasAr6a-EGFP-P2A-sombC1C2 (final concentration ~2×10^12^ GC/mL) mixed with AAV2/8 CaMKIIα-Flpo (final concentration ~0.5 – 1×10^10^ GC/mL) and CAV2-Cre (final concentration ~2.5 – 5×10^10^ GC/mL; CAV2-Cre was injected at ~2.5 ×10^10^ GC/mL for Figure 5 and ~5×10^10^ GC/mL for Figure 6). The virus for CA2/3 injection comprised AAV2/8 CaMKIIα-DIO-ChRmine-oScarlet-Kv2.1 (final concentration ~0.75 – 1×10^11^ GC/mL). Viruses were injected in the left hippocampal CA2/3 (500 nl, 45 – 60 nL/min, AP: −1.7 mm, ML: −2.0 mm, DV: −2.0 mm). For histology experiments characterizing the soma localization and axonal trafficking of ChRmine, to enrich the expression of ChRmine, the virus consisted of AAV2/8 CaMKIIα-DIO-ChRmine-oScarlet-Kv2.1 (final concentration ~7.5×10^10^ GC/mL) mixed with CKII(0.4)-Cre virus (final titer ~1×10^11^ GC/mL). In control conditions, the injected viruses consisted of AAV2/8 EF1α-DIO-oScarlet (final concentration ~7.5 – 10×10^10^ GC/mL) mixed with CKII(0.4)-Cre virus (final titer ~7.5×10^10^ GC/mL).

For experiments inhibiting CA2/3, the virus for CA2/3 injection consisted of AAV2/8 CaMKIIα-eHcKCR1–3.0-oScarlet-Kv2.1 (final concentration ~1 – 1.5×10^11^ GC/mL), injected into left hippocampal CA2/3 (300 nl, 45 – 60 nL/min, AP: −1.7 mm, ML: −2.0 mm, DV: −2.0 mm).

##### Cranial window surgery

The procedure for surgery and imaging in CA1 was based on the protocol from Dombeck et al.^77^ 8–12-week-old C57BL/6 mice (male and female) were deeply anesthetized with 2% isoflurane and maintained with ~1% isoflurane throughout the surgery. Before the start of surgery, animals were subcutaneously administered buprenorphine sustained release (SR) (0.3–1.0 mg/kg), 1 ml saline, carprofen (5 mg/kg) and dexamethasone (4.8 mg/kg). Throughout the surgery, eyes were kept moist using ophthalmic eye ointment. Body temperature was continuously monitored and maintained at 37 °C using a heating pad. The skull was exposed and thoroughly dried.

Virus was injected and then a 3 mm round craniotomy (centered at AP: −2 mm, ML: 2 mm) was opened using a biopsy punch (Miltex). The dura was then gently removed, and the overlying cortex was aspirated using a blunt aspiration needle under constant irrigation with cold PBS. The center region of the external capsule was removed to expose hippocampal CA1. A cannula window was prepared prior to the surgery and comprised a 1.5 mm long stainless steel tube (3 mm outer diameter, MicroGroup) and 3 mm round #1 cover glass (Harvard Apparatus) glued together with UV curable adhesive (Norland Products, NOA 81). Once bleeding stopped, the cannula was then lowered onto the CA1 surface until the window touched the tissue. The remaining outer surface of the cannula was sealed to the exposed skull with cyanoacrylate adhesive and dental cement that was dyed black using black ink (C&B metabond, Parkell, No. 242–3200). Finally, a stainless steel head plate was fixed onto the exposed skull. Animals were placed on a warming blanket to recover. Animals were typically active within 20 min and returned to their home cage for recovery. To avoid damage to the implant, mice were housed in separate cages. Mice were monitored for the next several days and given additional carprofen and buprenorphine if they showed signs of discomfort or infection. Mice were allowed to recover for ~7 days before beginning water restriction and VR training.

##### Fiber implantation

To avoid mutual obstruction between the fiber optics and the objective, optical fibers were implanted at 45 degrees. Specifically, 200 μm core diameter, 0.39 NA, 3 mm optical fibers (Thorlabs, FT200EMT) were implanted at 45 degrees over CA2/3 (−1.5 mm AP, −2.95 mm ML, −1.4 mm DV). Histology confirmed that the fibers were over the desired brain region.

#### Histology and confocal imaging

##### Histology

For histological analysis, injected mice and mice with cranial window and fiber implantation were transcardially perfused with ice cold PBS, immediately followed by perfusion of 4% paraformaldehyde (PFA). Brains were fixed overnight at 4 °C in PFA, then transferred to 30% sucrose/PBS solution. Coronal sections of either 100 mm (for in situ) or 150 μm (for regular histology) prepared using a vibratome (Leica) were collected and slice were stored in PBS. For nuclei staining, slices were incubated for 30 min with 4′,6-diamidino-2-phenylindol (DAPI) at 5 μg/mL, washed in PBST (0.1% Triton-X100) for 2 – 3 times, and then imaged.

##### In situ *hybridization*

Protocols for *in situ* RNA detection, probes for VGAT and dye-conjugated hairpins (B1–647, B5–546) were as previously described.^71^ Briefly, hybridizations with split probes were performed overnight in HCR hybridization buffer (Molecular Instruments) at 5 nM probe concentration. The next day, slices were washed three times in HCR wash buffer at 37 °C and then two times in 5× saline-sodium citrate with 0.1% Triton-X100 (SSCT) at room temperature, 20 min each, and then incubated in HCR amplification buffer. During this time, dye-conjugated hairpins were heated to 95 °C for 30 sec then snap-cooled on ice. Hairpin amplification was performed by incubating slices in 200 μL amplification buffer with B1 and B5 probes at concentrations of 240 nM overnight in the dark. Samples were stained with DAPI, washed three times with 5× SSCT for 30 min each and then imaged.

##### Immunohistochemistry

Brain was harvested as above and coronal sections of 60 μm were prepared with a freezing microtome (Leica) and stored in a cryoprotectant solution (25% glycerol, 30% ethylene glycol, in PBS). Before staining, slices were washed in PBS. Sections were blocked in 5% normal donkey serum (Jackson ImmunoResearch) in PBST for 60 min at room temperature followed by incubation with primary antibody: mouse anti-HA (1:500; ThermoFisher, A26183) overnight at 4 °C. Sections were subsequently washed three times in PBST (5 min each) and then transferred to a secondary antibody solution, donkey anti-Mouse 647 (1:500; ThermoFisher, A31571) for 3 h at room temperature. Samples were stained with DAPI, washed three times with PBST for 5 min each, and then imaged. Confocal fluorescence imaging was performed on an Olympus FV3000 confocal microscope.

#### VR design

VR setup was designed based on https://github.com/HarveyLab/mouseVR with a few modifications. ViRMEn^50^ software (Virtual Reality Mouse Engine, https://pni.princeton.edu/pni-software-tools/virmen) was used to design and implement the VR environments via a dedicated PC. Virtual environments were projected from a laser projector (LaserBeamPro C200) onto a parabolic screen that surrounded the mouse. Animals were head-fixed and ran on an 8-inch-diameter styrofoam ball which was fixed in one axis. The virtual environment was updated following movements of the animal, measured by two optical mouse sensors underneath the ball. Sucrose water rewards were given at the end of the track.

A National Instruments DAQ (NI cDAQ-9178) was used to interface with the VR setup. Using ViRMEn and the NI-DAQ, behavioral data, such as environment position, were used to compute custom gate signals, which were written to a NI-DAQ digital or analog output channel and used to synchronize stimulation with specific epochs in the behavior. For synchronization between behavioral data and voltage-imaging acquisition, TTL pulses were sent to the voltage imaging DAQ via the VR NI-DAQ at each trial start and end event, and computer timestamps were recorded on the behavior and voltage imaging computers. In the meantime, other real time information about behavior such as solenoid control signals and ball rotational velocities were also copied to the voltage imaging DAQ and computer.

#### Behavioral training

After cranial window surgery, mice were allowed to recover for ~7 days before beginning water restriction and VR training. To motivate mice to run, mice were placed on a water schedule in which they received 1 mL of water per day. Body weights were checked to ensure mice reached ~80–85% of their pre-water-restriction weight and they were given enough water to maintain this weight. After ~5 days of water scheduling, behavior training began. In each training session, mice were placed on the training VR apparatus with the head fixed in place, centered over the middle of the styrofoam ball with the headplate ~2.54 cm from the top of the ball.

The lick spout to deliver sucrose water reward (0.1g/mL sucrose) was positioned in front of the mouse’s mouth. The lick spout consisted of a feeding tube (Fine science tools, 18061–10) connected to a gravity-fed water line with an in-line solenoid valve (The lee company, LHDA0533115H). The solenoid valve was controlled using the National Instruments DAQ. The water delivery system was calibrated to deliver ~4 – 5 μL of liquid per drop. For lick detection, a wire was soldered to the feeding tube, and the capacitance of the feeding tube was sensed using an RC circuit. The metal headplate holder was grounded to the same apacitive-sensing circuit. The licks were read in as an analog signal through the National Instruments DAQ.

Mice were trained to run along the virtual track. The virtual track was 180 cm long, measured as the number of rotations of the ball to move from one end of the track to the other times the circumference of the ball. The virtual environment was designed as in previous studies^42^; the track had short proximal walls with different textures for each third of the track (0–60 cm: black rings, 61–120 cm: vertical white and black stripes, 121–180 cm: stars). Distal walls were positioned at 60 cm (gray bricks) and 120 cm (green crosses). The floor at the end of the track was green to mark reward zones. The rest of the floor was black throughout the track. The sucrose water rewards were given at the end of the track. Inter-trial interval (ITI) was set pseudorandomly between 1 – 3 sec. To engage the animals more, rewards were omitted on 20% of trials (Figure 1C). Mice were trained until the number of trials completed per day reach ~ 250 – 300 trials for 2 – 3 days. Training typically took ~7 – 14 days.

#### *In vivo* voltage imaging

Before imaging sessions started, animals were trained for at least two sessions on the experimental VR rig integrated with the optical system. Trained mice were imaged 1–3 months after virus injection, cranial window and fiber implantation surgeries. A typical imaging session lasted 1 – 2 hours. Recordings targeting hippocampal CA1 neurons *in vivo* were performed at a depth of 100 – 250 μm and typically targeted 1 – 10 neurons simultaneously. For characterizing place cells without optogenetic stimulation (Figure 1), in each imaging session, multiple FOVs were imaged at 1 kHz. For each FOV, a 30 sec protocol of continuous imaging was repeated 4 – 10 times.

#### Closed-loop optogenetic stimulation

For closed-loop optogenetic stimulation experiments, voltage imaging was performed as above (*in vivo* voltage imaging). Using ViRMEn and the NI-DAQ on the VR set-up, ongoing behavior of the environment position was used to compute custom gate signals, which were written to the NI-DAQ digital output channel to control optogenetic stimulation. A pixel bitmap was preloaded onto and projected from the DMD for targeted optogenetic stimulation. During each session with closed-loop optogenetic stimulation, the animal initially ran 30 trials over about 5 mins (defining the Pre epoch). After this baseline epoch, 300 ms targeted optogenetic stimulation were delivered upon crossing the 90 cm point of the virtual track (20 trials, ~3 – 4 mins defining the Stim epoch). This was followed by VR trials without stimulation for 10 – 30 mins (~50 – 200 trials, defining the Post epoch).

Endogenous background from the brain arising from optogenetic-wavelength illumination contributed to a small step-like increase of fluorescence in the GEVI channel. For optogenetic inhibition of CA2/3, using ViRMEn and the NI-DAQ on the VR set-up, ongoing behavior of each VR trial was used to compute custom gate signals, which were written to the NI-DAQ digital output channel to control optogenetic inhibition. Optogenetic inhibition of CA2/3 was delivered across the whole virtual space during the ‘‘Stim with CA2/3 inh’’ epoch for 10 trials.

### QUANTIFICATION AND STATISTICAL ANALYSIS

Data were analyzed with custom code written in MATLAB. Voltage imaging data were analyzed as previously described.^36^

#### Corrections for photobleaching and motion artifacts

Movies were first corrected for motion using the NoRMCorre algorithm.^72^ Movies were then corrected for photobleaching by dividing the movie by an exponential fit of the mean fluorescence.

#### Image segmentation and waveform extraction

We divided the movie into sub-movies comprising single cells and performed activity-based image segmentation separately in each sub-movie. Whereas correlations often arose between subthreshold voltages and out-of-focus background, we assumed that spiking was not correlated with background, and furthermore that the spatial footprint associated with spiking would be the same as for true subthreshold dynamics. For segmentation purposes, we first removed subthreshold signals via a 100 Hz high-pass filter; movies were then segmented semi-automatically using activity-based segmentation algorithms. Principal components analysis was followed by time-domain independent components analysis (PCA/ICA).^78^ The spatial masks from PCA/ICA were then applied to the original movies without high-pass filtering to extract fluorescence traces.

#### Spike finding, spike removal, and scaling of fluorescence recordings

A simple threshold-and-maximum procedure was applied for spike detection. Fluorescence traces were first high-pass filtered, and initial threshold was set at 4x noise level. This threshold was then manually adjusted. For spike removal, spikes were digitally removed and replaced with linear interpolation of the surrounding data. Linear interpolations were performed between data-points 3 ms before and 6 ms after action potential peak. To compute ΔV_m_, fluorescence values were low-passed filtered (session below, ramping subthreshold membrane potential) and compared with mean fluorescence of the entire trace after spike removal (including a segment of the inter-trial interval). All fluorescence signals were normalized to spike height.

#### Spike detection: false-positive and false-negative rates

For an SNR of 8.8 and a spike-detection threshold set at 4σ about the baseline noise, the false-positive rate is calculated as the probability that samples from a Gaussian distribution lie more than 4σ above the mean. This probability is (1-p_4_)/2*1 kHz*30 s, where p_4_ = 0.999937. The false-negative rate is calculated as the probability that spike height values fall more than 4.8s below the mean (7.9333 × 10^−7^).

#### Firing rate maps

To create firing rate maps, we divided the virtual linear track into 80 bins (2.25 cm each) and calculated the firing rate as the total number of spikes in a bin divided by the total amount of time spent in a bin. The maps were smoothed using a five point Gaussian window with standard deviation of one. Periods in which the mouse ran slower than 5 cm/s were removed from the analysis. Cells with VR trials less than 5 were discarded for place cell analysis. Cells with an overall mean firing rate less than 0.1 Hz were discarded for place cell analysis.

#### Place cell identification with spatial information metric

Place cells were identified using a previously published spatial information (SI) metric^46^:

$$SI=\sum_{i=1}^{n_{bin}}\;p_{i}\lambda_{i}{log}_{2}\frac{\lambda_{i}}{\lambda }$$

where i is the spatial bin, $n_{\text{bin}}$ is the number of bins, $p_{i}$ is the fractional occupancy of bin $i,\;\lambda_{i}$ is the mean firing rate in position bin i,λ is the overall mean firing rate of the cell. To determine the significance of the SI value, we performed a bootstrap shuffle test. On each shuffling iteration, we circularly permuted the time series of the firing rate relative to the position trace within each trial and recalculated the SI for the shuffled data. Shuffling was performed 1,000 times for each cell, and cells that exceeded over 95% of the permutations were determined to be significant place cells.

#### Place field identification

To identify place fields, we found groups of adjacent bins with firing rates greater than 0.25 times the rate in the peak bin. We selected only those fields that were larger than 4 bins (9 cm) in length, had mean in-field firing rates of greater than 1.5 Hz, and had mean in-field firing rates more than 2.5 times larger than the mean out-of-field firing rate.

#### Plateau-driven complex spikes

To identify plateau-driven complex spikes, we smoothed the action potential-deleted traces with a moving-average filter (20 ms window). Events were detected from the smoothed trace by threshold crossing at 40% of spike height. Only events that lasted longer than 10 ms and rose atop spiking bursts were considered as plateau events. All fluorescence signals were normalized to spike height. To quantify the complex spike rate as a function of position along the virtual track, we divided the virtual linear track into 80 bins (2.25 cm each) and calculated the event rate as the total number of events in a bin divided by the total amount of time spent in a bin.

#### Ramping subthreshold membrane potential

To analyze subthreshold ramping membrane potentials, we low-pass filtered (<3 Hz) the action potential-deleted traces using an FIR filter with a 200 ms Hamming window. All fluorescence signals were normalized to spike height. To quantify the ramping subthreshold membrane potential as a function of position along the virtual track, we divided the virtual linear track into 80 bins (2.25 cm each) and then created a map of membrane potential values by grouping the membrane potentials into 80 spatial bins along the track and calculating an average membrane potential for each bin. The membrane potential map was smoothed using a five point Gaussian window with a standard deviation of one. Periods in which the mouse ran slower than 5 cm/s were removed from the analysis.

#### Theta oscillation

To analyze subthreshold theta oscillations, we band-pass filtered (6–10 Hz) the raw action potential-deleted traces using an FIR filter with a 200-ms Hamming window and derived the amplitude vector using the Hilbert transform. All fluorescence signals were normalized to spike height. To quantify the theta-rhythm amplitude as a function of position along the virtual track, we divided the virtual linear track into 80 bins (2.25 cm each) and then created a map of theta-rhythm amplitudes by grouping the amplitude into 80 spatial bins along the track and calculating an average amplitude for each bin. The theta-rhythm amplitude map was smoothed using a five point Gaussian window with a standard deviation of one. Periods in which the mouse ran slower than 5 cm/s were removed from the analysis.

#### Baseline to spike-initiation threshold

All fluorescence signals were normalized to spike height. To quantify the baseline to spike-initiation threshold, we calculated the derivative of the spike-triggered average waveform (dSTA/dt), and set threshold to the value at which dSTA/dt crossed 0.1 spike height/sec.

#### CA2/3 stimulation evoked spiking

A spike was classified as ‘evoked’ if it occurred within 26 ms of onset of the CA2/3 stimulus^36,40,56^; setting this value included consideration that the CA2/3 stimulus was 20 ms repeated at 1 Hz, that spontaneous firing rate in CA1 is low (about 2 Hz; Figure S5), and that several events are sequentially involved between light pulse initiation and postsynaptic spiking (optogenetic stimulus-induced depolarization leading to spiking in presynaptic cells, synaptic transmission, and synaptic depolarization leading to spiking in postsynaptic cells.^36,40,56^

#### Classification of synaptic potentials in response to CA2/3 stimulation

We quantified the evoked spiking events and calculated area under curve (AUC) of the fluorescence trace in response to CA2/3 stimulus. Cells with no evoked spikes detected and AUC < 0 were classified as having inhibitory inputs. Cells with more than one evoked spike out of 12 trials or AUC > 0 were classified as receiving excitatory inputs.

#### Statistics

All error ranges represent standard error of the mean, unless otherwise specified. For the same neurons inside/outside of place field, before/after optogenetic stimulation, and before/after plasticity, the paired sample t-test was used. For two-sample comparisons of a single variable, student’s t-test was used. In cases where the underlying distributions were non-Gaussian, the Kruskal-Wallis test was used. Probabilities of the null hypothesis *p* < 0.05 were judged to be statistically significant.

## Supplementary Material

1

## Figures and Tables

**KEY RESOURCES TABLE:** 

REAGENT or RESOURCE	SOURCE	IDENTIFIER

Bacterial and virus strains		

AAV8_CaMKIIα-Flpo	Stanford Gene Vector and Virus Core	N/A
LZF2039CR AAV2/9_hSyn-fDIO-somQuasAr6a-EGFP-P2A-sombC1C2TG	Janelia Farm Vector Core	N/A
CAV2-Cre	CNRS Vector Core	N/A
AAV8_CaMKIIα-DIO-ChRmine-oScarlet-Kv2.1	Stanford Gene Vector and Virus Core	N/A
AAV9_CKII(0.4)-Cre	Addgene	105558-AAV9
AAV8_EF1α-DIO-oScarlet	Stanford Gene Vector and Virus Core	N/A
AAV8_CaMKIIα-eHcKCR1–3.0-oScarlet-Kv2.1	Stanford Gene Vector and Virus Core	N/A

Chemicals, peptides, and recombinant proteins		

VGAT probe, dye-conjugated hairpins (B1-647, B5-546)	Chen et al.^71^	N/A
HA Tag monoclonal antibody	ThermoFisher	cat# A26183; RRID: AB_10978021
Donkey anti-Mouse IgG (H±L) Highly Cross-Adsorbed Secondary Antibody, Alexa Fluor 647	ThermoFisher	cat# A31571; RRID: AB_162542

Experimental models: Organisms/strains		

C57BL/6 wild-type mice	Jackson Lab	Stock #000664
Ai14 transgenic mice	Jackson Lab	Stock #007914

Recombinant DNA		

LZF2039CR pAAV_hSyn-fDIO-somQuasAr6a-EGFP-P2A-sombC1C2TG	This work	Addgene 183537
pAAV_CaMKIIα-Flpo	This work	Addgene 183535
pAAV_CaMKIIα-DIO-ChRmine-oScarlet-Kv2.1	This work	Addgene 183536
pAAV_CaMKIIα-eHcKCR1–3.0-oScarlet-Kv2.1	This work	Addgene 195198

Software and algorithms		

Matlab R2015b – 2020a	Mathworks	https://www.mathworks.com/products/matlab.html
Labview	National Instrument	N/A
NoRMCorre	Pnevmatikakis and Giovannucci^72^	N/A
ViRMEn	Aronov and Tank^50^	https://pni.princeton.edu/pni-software-tools/virmen

Other		

Custom-designed microscope	This work	N/A
Virtual Reality Setup	Harvey et al.^42^	https://github.com/HarveyLab/mouseVR

Deposited data		

Voltage imaging data	DANDI Archive	N/A

## Highlights

- All-optical modulation/readout of membrane voltage in hippocampal plasticity (BTSP)
- Cellular-resolution all-optical measurements reveal synaptic potentiation in BTSP
- Cellular-resolution all-optical measurements reveal no excitability changes in BTSP
- Presynaptic (CA2/3) region activity required for full postsynaptic (CA1)-induced BTSP

## References

[1] Hebb DO (1949). Organization of Behavior (Wiley).

[2] Luo L (2020). Principles of Neurobiology (Garland Science).

[3] Bittner KC, Milstein AD, Grienberger C, Romani S, and Magee JC (2017). Behavioral time scale synaptic plasticity underlies CA1 place fields. Science 357, 1033–1036. 10.1126/science.aan3846. 28883072 PMC7289271

[4] Bear MF, and Malenka RC (1994). Synaptic plasticity: LTP and LTD. Curr. Opin. Neurobiol. 4, 389–399. 10.1016/0959-4388(94)90101-5. 7919934

[5] Huerta PT, and Lisman JE (1995). Bidirectional synaptic plasticity induced by a single burst during cholinergic theta oscillation in CA1 in vitro. Neuron 15, 1053–1063. 10.1016/0896-6273(95)90094-2. 7576649

[6] Deisseroth K, Bito H, and Tsien RW (1996). Signaling from synapse to nucleus: postsynaptic CREB phosphorylation during multiple forms of hippocampal synaptic plasticity. Neuron 16, 89–101. 10.1016/S0896-6273(00)80026-4. 8562094

[7] Bi GQ, and Poo MM (1998). Synaptic modifications in cultured hippocampal neurons: dependence on spike timing, synaptic strength, and postsynaptic cell type. J. Neurosci. 18, 10464–10472. 10.1523/JNEUROSCI.18-24-10464.1998. 9852584 PMC6793365

[8] Turrigiano GG, Leslie KR, Desai NS, Rutherford LC, and Nelson SB (1998). Activity-dependent scaling of quantal amplitude in neocortical neurons. Nature 391, 892–896. 10.1038/36103. 9495341

[9] Dudman JT, Tsay D, and Siegelbaum SA (2007). A role for synaptic inputs at distal dendrites: instructive signals for hippocampal long-term plasticity. Neuron 56, 866–879. 10.1016/J.NEURON.2007.10.020. 18054862 PMC2179894

[10] Deisseroth K, Bito H, Schulman H, and Tsien RW (1995). Synaptic plasticity: a molecular mechanism for metaplasticity. Curr. Biol. 5, 1334–1338. 10.1016/S0960-9822(95)00262-4. 8749377

[11] Bliss TVP, and Lømo T (1973). Long-lasting potentiation of synaptic transmission in the dentate area of the anaesthetized rabbit following stimulation of the perforant path. J. Physiol. 232, 331–356. 10.1113/jphysiol.1973.sp010273. 4727084 PMC1350458

[12] Graves AR, Roth RH, Tan HL, Zhu Q, Bygrave AM, Lopez-Ortega E, Hong I, Spiegel AC, Johnson RC, Vogelstein JT, (2021). Visualizing synaptic plasticity in vivo by large-scale imaging of endogenous ampa receptors. eLife 10, e66809. 10.7554/eLife.66809. 34658338 PMC8616579

[13] Kim JJ, and Fanselow MS (1992). Modality-specific retrograde amnesia of fear. Science 256, 675–677. 10.1126/science.1585183. 1585183

[14] Morris RGM, Garrud P, Rawlins JNP, and O’Keefe J (1982). Place navigation impaired in rats with hippocampal lesions. Nature 297, 681–683. 10.1038/297681a0. 7088155

[15] Morris RGM, Anderson E, Lynch GS, and Baudry M (1986). Selective impairment of learning and blockade of long-term potentiation by an N-methyl-D-aspartate receptor antagonist, AP5. Nature 319, 774–776. 10.1038/319774a0. 2869411

[16] Scoville WB, and Milner B (1957). Loss of recent memory after bilateral hippocampal lesions. J. Neurol. Neurosurg. Psychiatry 20, 11–21. 10.1136/JNNP.20.1.11. 13406589 PMC497229

[17] Robinson NTM, Descamps LAL, Russell LE, Buchholz MO, Bicknell BA, Antonov GK, Lau JYN, Nutbrown R, Schmidt-Hieber C, and Häusser M (2020). Targeted activation of hippocampal place cells drives memory-guided spatial behavior. Cell 183, 2041–2042. 10.1016/j.cell.2020.12.010. 33357402 PMC7773032

[18] O’Keefe J, and Dostrovsky J (1971). The hippocampus as a spatial map. Preliminary evidence from unit activity in the freely-moving rat. Brain Res. 34, 171–175. 10.1016/0006-8993(71)90358-1. 5124915

[19] Tsien JZ, Huerta PT, and Tonegawa S (1996). The essential role of hippocampal CA1 NMDA receptor-dependent synaptic plasticity in spatial memory. Cell 87, 1327–1338. 10.1016/S0092-8674(00)81827-9. 8980238

[20] Whitlock JR, Heynen AJ, Shuler MG, and Bear MF (2006). Learning induces long-term potentiation in the hippocampus. Science 313, 1093–1097. 10.1126/SCIENCE.1128134. 16931756

[21] Sheffield MEJ, Adoff MD, and Dombeck DA (2017). Increased prevalence of calcium transients across the dendritic arbor during place field formation. Neuron 96. 490–504.e5. 10.1016/J.NEURON.2017.09.029. 29024668 PMC5642299

[22] Bittner KC, Grienberger C, Vaidya SP, Milstein AD, Macklin JJ, Suh J, Tonegawa S, and Magee JC (2015). Conjunctive input processing drives feature selectivity in hippocampal CA1 neurons. Nat. Neurosci. 18, 1133–1142. 10.1038/nn.4062. 26167906 PMC4888374

[23] Zhao X, Wang Y, Spruston N, and Magee JC (2020). Membrane potential dynamics underlying context-dependent sensory responses in the hippocampus. Nat. Neurosci. 23, 881–891. 10.1038/s41593-020-0646-2. 32451487

[24] Pignatelli M, Ryan TJ, Roy DS, Lovett C, Smith LM, Muralidhar S, and Tonegawa S (2019). Engram cell excitability state determines the efficacy of memory retrieval. Neuron 101. 274–284.e5. 10.1016/j.neuron.2018.11.029. 30551997

[25] Lee JS, Briguglio JJ, Cohen JD, Romani S, and Lee AK (2020). The statistical structure of the hippocampal code for space as a function of time, context, and value. Cell 183. 620–635.e22. 10.1016/J.CELL.2020.09.024. 33035454

[26] Alejandre-García T, Kim S, Pérez-Ortega J, and Yuste R (2022). Intrinsic excitability mechanisms of neuronal ensemble formation. eLife 11, e77470. 10.7554/eLife.77470. 35506662 PMC9197391

[27] Petersen CCH (2017). Whole-cell recording of neuronal membrane potential during behavior. Neuron 95, 1266–1281. 10.1016/j.neuron.2017.06.049. 28910617

[28] Pala A, and Petersen CCH (2015). In vivo measurement of cell-type-specific synaptic connectivity and synaptic transmission in Layer 2/3 mouse barrel cortex. Neuron 85, 68–75. 10.1016/J.NEURON.2014.11.025. 25543458 PMC4305188

[29] Geiller T, Sadeh S, Rolotti S.V.v., Blockus H, Vancura B, Negrean A, Murray AJ, Rózsa B, Polleux F, Clopath C, and Losonczy A (2022). Local circuit amplification of spatial selectivity in the hippocampus. Nature 601, 105–109. 10.1038/s41586-021-04169-9. 34853473 PMC9746172

[30] Rolotti S.V.v., Ahmed MS, Szoboszlay M, Geiller T, Negrean A, Blockus H, Gonzalez KC, Sparks FT, Solis Canales AS, Tuttman AL, (2022). Local feedback inhibition tightly controls rapid formation of hippocampal place fields. Neuron 110. 783–794.e6. 10.1016/J.NEURON.2021.12.003. 34990571 PMC8897257

[31] Rickgauer JP, Deisseroth K, and Tank DW (2014). Simultaneous cellular-resolution optical perturbation and imaging of place cell firing fields. Nat. Neurosci. 17, 1816–1824. 10.1038/nn.3866. 25402854 PMC4459599

[32] Adam Y, Kim JJ, Lou S, Zhao Y, Xie ME, Brinks D, Wu H, Mostajo-Radji MA, Kheifets S, Parot V, (2019). Voltage imaging and optogenetics reveal behaviour-dependent changes in hippocampal dynamics. Nature 569, 413–417. 10.1038/s41586-019-1166-7. 31043747 PMC6613938

[33] Piatkevich KD, Bensussen S, Tseng H-A, Shroff SN, Lopez-Huerta VG, Jung EE, Shemesh OA, Straub C, Gritton HJ, (2019). Population imaging of neural activity in awake behaving mice. Nature 574, 413–417. 10.1038/s41586-019-1641-1. 31597963 PMC6858559

[34] Abdelfattah AS, Kawashima T, Singh A, Novak O, Liu H, Shuai Y, Huang Y-C, Campagnola L, Seeman SC, Yu J, (2019). Bright and photostable chemigenetic indicators for extended in vivo voltage imaging. Science 365, 699–704. 10.1126/science.aav6416. 31371562

[35] Villette V, Chavarha M, Dimov IK, Bradley J, Pradhan L, Mathieu B, Evans SW, Chamberland S, Shi D, Yang R, (2019). Ultrafast two-photon imaging of a high-gain voltage indicator in awake behaving mice. Cell 179. 1590–1608.e23. 10.1016/j.cell.2019.11.004. 31835034 PMC6941988

[36] Fan LZ, Kheifets S, Böhm UL, Wu H, Piatkevich KD, Xie ME, Parot V, Ha Y, Evans KE, Boyden ES, (2020). All-optical electrophysiology reveals the role of lateral inhibition in sensory processing in cortical Layer 1. Cell 180. 521–535.e18. 10.1016/J.CELL.2020.01.001. 31978320 PMC7259440

[37] Böhm UL, Kimura Y, Kawashima T, Ahrens MB, Higashijima S, Engert F, and Cohen AE (2022). Voltage imaging identifies spinal circuits that modulate locomotor adaptation in zebrafish. Neuron 110, e4. 10.1016/J.NEURON.2022.01.001. PMC8989672 35104451

[38] Chien MP, Brinks D, Testa-Silva G, Tian H, Phil Brooks F, Adam Y, Bloxham B, Gmeiner B, Kheifets S, and Cohen AE (2021). Photoactivated voltage imaging in tissue with an archaerhodopsin-derived reporter. Sci. Adv. 7, eabe3216. 10.1126/SCIADV.ABE3216. 33952514 PMC8099184

[39] Kralj JM, Douglass AD, Hochbaum DR, Maclaurin D, and Cohen AE (2011). Optical recording of action potentials in mammalian neurons using a microbial rhodopsin. Nat. Methods 9, 90–95. 10.1038/nmeth.1782. 22120467 PMC3248630

[40] Fan LZ, Nehme R, Adam Y, Jung ES, Wu H, Eggan K, Arnold DB, and Cohen AE (2018). All-optical synaptic electrophysiology probes mechanism of ketamine-induced disinhibition. Nat. Methods 15, 823–831. 10.1038/s41592-018-0142-8. 30275587 PMC6204345

[41] Tian H, Davis HC, Wong-Campos JD, Fan LZ, Gmeiner B, Begum S, Werley CA, Borja GB, Upadhyay H, Shah H, (2022). All-optical electrophysiology with improved genetically encoded voltage indicators reveals interneuron network dynamics in vivo. Preprint at bioRxiv. 10.1101/2021.11.22.469481.

[42] Harvey CD, Collman F, Dombeck DA, and Tank DW (2009). Intracellular dynamics of hippocampal place cells during virtual navigation. Nature 461, 941–946. 10.1038/nature08499. 19829374 PMC2771429

[43] Rajasethupathy P, Sankaran S, Marshel JH, Kim CK, Ferenczi E, Lee SY, Berndt A, Ramakrishnan C, Jaffe A, Lo M, (2015). Projections from neocortex mediate top-down control of memory retrieval. Nature 526, 653–659. 10.1038/nature15389. 26436451 PMC4825678

[44] Berndt A, Lee SY, Ramakrishnan C, and Deisseroth K (2014). Structure-guided transformation of channelrhodopsin into a light-activated chloride channel. Science 344, 420–424. 10.1126/SCIENCE.1252367. 24763591 PMC4096039

[45] Tee BCK, Chortos A, Berndt A, Nguyen AK, Tom A, McGuire A, Lin ZC, Tien K, Bae WG, Wang H, (2015). A skin-inspired organic digital mechanoreceptor. Science 350, 313–316. 10.1126/SCIENCE.AAA9306. 26472906

[46] Skaggs WE, Mcnaughton BL, Gothard KM, and Markus EJ (1992). An information-theoretic approach to deciphering the hippocampal code. Adv. Neural Inf. Process. Syst. 5, 1030–1037.

[47] Epsztein J, Brecht M, and Lee AK (2011). Intracellular determinants of hippocampal CA1 place and silent cell activity in a novel environment. Neuron 70, 109–120. 10.1016/J.NEURON.2011.03.006. 21482360 PMC3221010

[48] Lee D, Lin BJ, and Lee AK (2012). Hippocampal place fields emerge upon single-cell manipulation of excitability during behavior. Science 337, 849–853. 10.1126/science.1221489. 22904011

[49] Grienberger C, Milstein AD, Bittner KC, Romani S, and Magee JC (2017). Inhibitory suppression of heterogeneously tuned excitation enhances spatial coding in CA1 place cells. Nat. Neurosci. 20, 417–426. 10.1038/nn.4486. 28114296

[50] Aronov D, and Tank DW (2014). Engagement of neural circuits underlying 2D spatial navigation in a rodent virtual reality system. Neuron 84, 442–456. 10.1016/J.NEURON.2014.08.042. 25374363 PMC4454359

[51] Stachenfeld KL, Botvinick MM, and Gershman SJ (2017). The hippocampus as a predictive map. Nat. Neurosci. 20, 1643–1653. 10.1038/nn.4650. 28967910

[52] Xu X, Sun Y, Holmes TC, and López AJ (2016). Noncanonical connections between the subiculum and hippocampal CA1. J. Comp. Neurol. 524, 3666–3673. 10.1002/CNE.24024. 27150503 PMC5050062

[53] Davoudi H, and Foster DJ (2019). Acute silencing of hippocampal CA3 reveals a dominant role in place field responses. Nat. Neurosci. 22, 337–342. 10.1038/s41593-018-0321-z. 30664772 PMC6387637

[54] Nakashiba T, Young JZ, McHugh TJ, Buhl DL, and Tonegawa S (2008). Transgenic inhibition of synaptic transmission reveals role of CA3 output in hippocampal learning. Science 319, 1260–1264. 10.1126/SCIENCE.1151120. 18218862

[55] Finnerty GT, and Jefferys JGR (1993). Functional connectivity from ca3 to the ipsilateral and contralateral ca1 in the rat dorsal hippocampus. Neuroscience 56, 101–108. 10.1016/0306-4522(93)90566-X. 8232909

[56] Hage TA, Bosma-Moody A, Baker CA, Kratz MB, Campagnola L, Jarsky T, Zeng H, and Murphy GJ (2022). Synaptic connectivity to L2/3 of primary visual cortex measured by two-photon optogenetic stimulation. eLife 11, e71103. 10.7554/eLife.71103. 35060903 PMC8824465

[57] Pouille F, and Scanziani M (2004). Routing of spike series by dynamic circuits in the hippocampus. Nature 429, 717–723. 10.1038/nature02615. 15170216

[58] Glickfeld LL, and Scanziani M (2006). Distinct timing in the activity of cannabinoid-sensitive and cannabinoid-insensitive basket cells. Nat. Neurosci. 9, 807–815. 10.1038/nn1688. 16648849 PMC3509385

[59] Govorunova EG, Gou Y, Sineshchekov OA, Li H, Lu X, Wang Y, Brown LS, St-Pierre F, Xue M, and Spudich JL (2022). Kalium channelrhodopsins are natural light-gated potassium channels that mediate optogenetic inhibition. Nat. Neurosci. 25, 967–974. 10.1038/s41593-022-01094-6. 35726059 PMC9854242

[60] Takahashi H, and Magee JC (2009). Pathway interactions and synaptic plasticity in the dendritic tuft regions of CA1 pyramidal neurons. Neuron 62, 102–111. 10.1016/J.NEURON.2009.03.007. 19376070

[61] Morgan PJ, Bourboulou R, Filippi C, Koenig-Gambini J, and Epsztein J (2019). Kv1.1 contributes to a rapid homeostatic plasticity of intrinsic excitability in CA1 pyramidal neurons in vivo. eLife 8, e49915. 10.7554/eLife.49915. 31774395 PMC6881145

[62] Cai DJ, Aharoni D, Shuman T, Shobe J, Biane J, Song W, Wei B, Veshkini M, La-Vu M, Lou J, (2016). A shared neural ensemble links distinct contextual memories encoded close in time. Nature 534, 115–118. 10.1038/nature17955. 27251287 PMC5063500

[63] Packer AM, Russell LE, Dalgleish HWP, and Häusser M (2015). Simultaneous all-optical manipulation and recording of neural circuit activity with cellular resolution in vivo. Nat. Methods 12, 140–146. 10.1038/nmeth.3217. 25532138 PMC4933203

[64] Sridharan S, Gajowa MA, Ogando MB, Jagadisan UK, Abdeladim L, Sadahiro M, Bounds HA, Hendricks WD, Turney TS, Tayler I, (2022). High-performance microbial opsins for spatially and temporally precise perturbations of large neuronal networks. Neuron 110. 1139–1155.e6. 10.1016/j.neuron.2022.01.008. 35120626 PMC8989680

[65] Dong C, Madar AD, and Sheffield MEJ (2021). Distinct place cell dynamics in CA1 and CA3 encode experience in new environments. Nat. Commun. 12, 2977. 10.1038/s41467-021-23260-3. 34016996 PMC8137926

[66] Terada S, Geiller T, Liao Z, O’Hare J, Vancura B, and Losonczy A (2022). Adaptive stimulus selection for consolidation in the hippocampus. Nature 601, 240–244. 10.1038/S41586-021-04118-6. 34880499 PMC9380538

[67] Grienberger C, and Magee JC (2022). Entorhinal cortex directs learning-related changes in CA1 representations. Nature 611, 554–562. 10.1038/S41586-022-05378-6. 36323779 PMC9668747

[68] Yap EL, Pettit NL, Davis CP, Nagy MA, Harmin DA, Golden E, Dagliyan O, Lin C, Rudolph S, Sharma N, (2021). Bidirectional perisomatic inhibitory plasticity of a Fos neuronal network. Nature 590, 115–121. 10.1038/s41586-020-3031-0. 33299180 PMC7864877

[69] Wilson RI, and Nicoll RA (2001). Endogenous cannabinoids mediate retrograde signalling at hippocampal synapses. Nature 410, 588–592. 10.1038/35069076. 11279497

[70] McKenzie S, Huszár R, English DF, Kim K, Christensen F, Yoon E, and Buzsáki G (2021). Preexisting hippocampal network dynamics constrain optogenetically induced place fields. Neuron 109. 1040–1054. e7. 10.1016/J.NEURON.2021.01.011. 33539763 PMC8095399

[71] Chen R, Gore F, Nguyen QA, Ramakrishnan C, Patel S, Kim SH, Raffiee M, Kim YS, Hsueh B, Krook-Magnusson E, (2021). Deep brain optogenetics without intracranial surgery. Nat. Biotechnol. 39, 161–164. 10.1038/s41587-020-0679-9. 33020604 PMC7878426

[72] Pnevmatikakis EA, and Giovannucci A (2017). NoRMCorre: an online algorithm for piecewise rigid motion correction of calcium imaging data. J. Neurosci. Methods 291, 83–94. 10.1016/j.jneumeth.2017.07.031. 28782629

[73] Marshel JH, Kim YS, Machado TA, Quirin S, Benson B, Kadmon J, Raja C, Chibukhchyan A, Ramakrishnan C, Inoue M, (2019). Cortical layer–specific critical dynamics triggering perception. Science 365, eaaw5202. 10.1126/science.aaw5202. 31320556 PMC6711485

[74] Kato HE, Kamiya M, Sugo S, Ito J, Taniguchi R, Orito A, Hirata K, Inutsuka A, Yamanaka A, Maturana AD, (2015). Atomistic design of microbial opsin-based blue-shifted optogenetics tools. Nat. Commun. 6, 7177. 10.1038/ncomms8177. 25975962 PMC4479019

[75] Gradinaru V, Zhang F, Ramakrishnan C, Mattis J, Prakash R, Diester I, Goshen I, Thompson KR, and Deisseroth K (2010). Molecular and cellular approaches for diversifying and extending optogenetics. Cell 141, 154–165. 10.1016/j.cell.2010.02.037. 20303157 PMC4160532

[76] Foutz TJ, Arlow RL, and McIntyre CC (2012). Theoretical principles underlying optical stimulation of a channelrhodopsin-2 positive pyramidal neuron. J. Neurophysiol. 107, 3235–3245. 10.1152/JN.00501.2011. 22442566 PMC3378402

[77] Dombeck DA, Harvey CD, Tian L, Looger LL, and Tank DW (2010). Functional imaging of hippocampal place cells at cellular resolution during virtual navigation. Nat. Neurosci. 13, 1433–1440. 10.1038/nn.2648. 20890294 PMC2967725

[78] Mukamel EA, Nimmerjahn A, and Schnitzer MJ (2009). Automated analysis of cellular signals from large-scale calcium imaging data. Neuron 63, 747–760. 10.1016/j.neuron.2009.08.009. 19778505 PMC3282191

