# Supplementary material for: All-optical physiology resolves a synaptic basis for behavioral timescale plasticity
Source: Cell. Author manuscript; Available in PMC 2023 Jul 7. (PMC10327443; doi:10.1016/j.cell.2022.12.035)
Supplement: 1 [file NIHMS1907967-supplement-1.pdf]

Supplemental figures

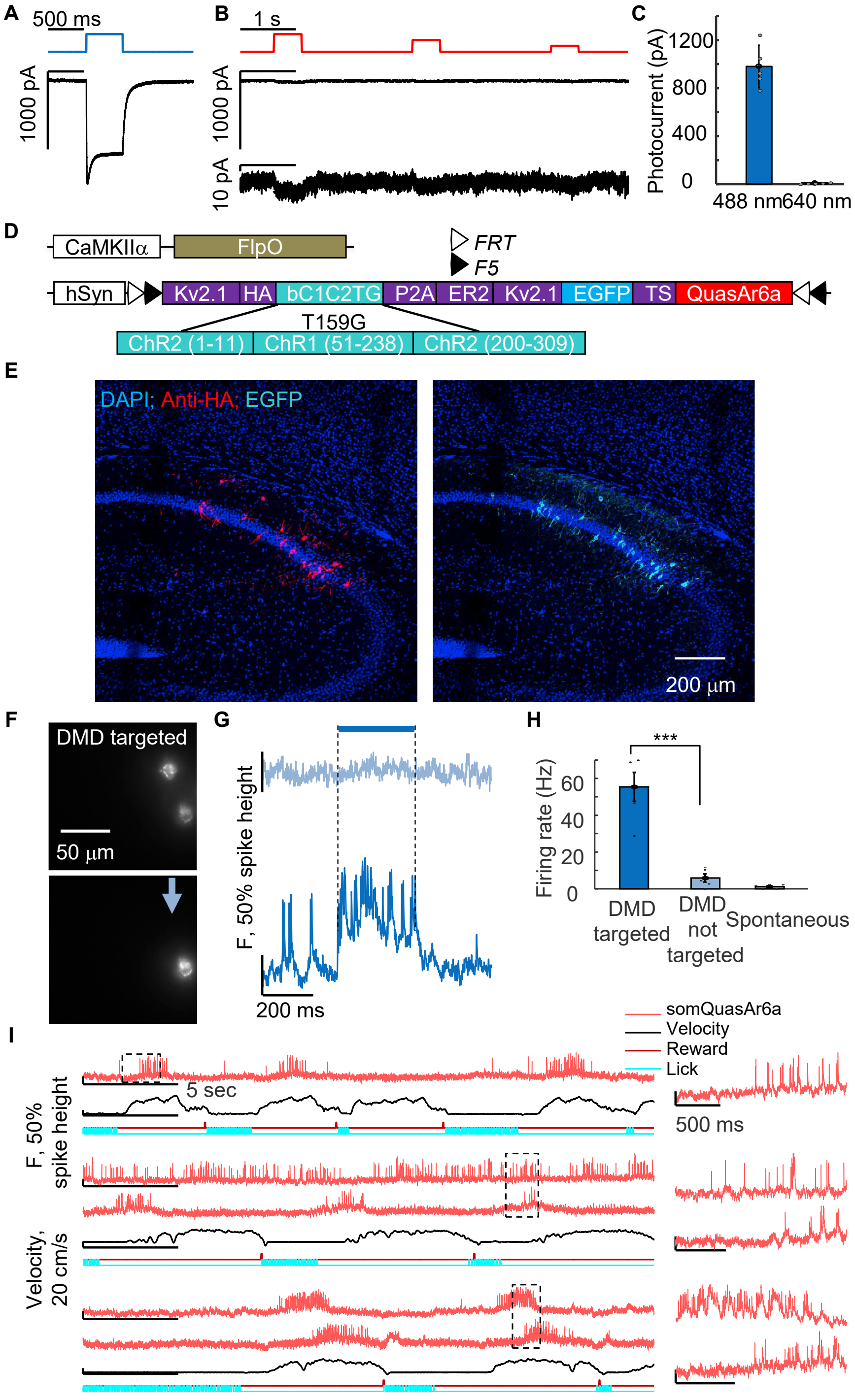

Figure S1. Characterization of somQuasAr6a-P2A-sombC1C2TG, related to Figures 1, 2, and 3

(A) Representative photocurrent of bC1C2TG in response to blue illumination (500 ms pulses, 488 nm, 1 mW/mm^2).

(B) Representative photocurrent of bC1C2TG in response to red illumination (500 ms pulses, 639 nm, 9–4.8–2.4 W/mm^2). Top: same current scale as in (A), bottom: expanded current scale.

(C) Comparison of mean photocurrent for blue (980 ± 177 pA) and red (11 ± 3 pA) illumination (mean ± SD, n = 5 cells. Intensity was 9 W/mm^2 for red).

(legend continued on next page)

(D) Schematic of the construct for viral Flp-dependent co-expression of somQuasAr6a and sombC1C2TG.

(E) Confocal images of fixed brain slices showing somQuasAr6a (cyan, EGFP was fused to somQuasAr6a) and sombC1C2TG (red, HA was fused to sombC1C2TG).

(F) Two epifluorescence images of the same field of view *in vivo*. Fluorescence in the EGFP channel showing DMD-patterned blue illumination targeted to the somata of two neurons (top) expressing somQuasAr6a-EGFP-P2A-sombC1C2TG and targeted to soma of only the bottom neuron (bottom). Blue arrow: top neuron displays little fluorescence, revealing minimal scattering of blue light.

(G) Optically recorded spiking of neurons with DMD-targeted illumination (bottom), and surrounding neurons with DMD not targeted (top).

(H) Comparison of spike rates for cells among DMD-targeted (55.5 ± 7.9 Hz), DMD-not-targeted (5.8 ± 2.2 Hz), and no (1.2 ± 0.5 Hz) blue illumination conditions (mean ± SEM, $p = 3 \times 10^{-4}$, two-tailed t test, blue light intensity 25 mW/mm^2, n = 6 cells).

(I) Voltage imaging of CA1 cells during VR behavior. Red: single-trial unfiltered fluorescence traces of somQuasAr6a recorded at 1 kHz. Black: mouse running velocity. Dark red line with ticks at bottom: reward delivery. Cyan: detected licking periods (reward retrieval). Right: magnified views of the boxed regions at left.

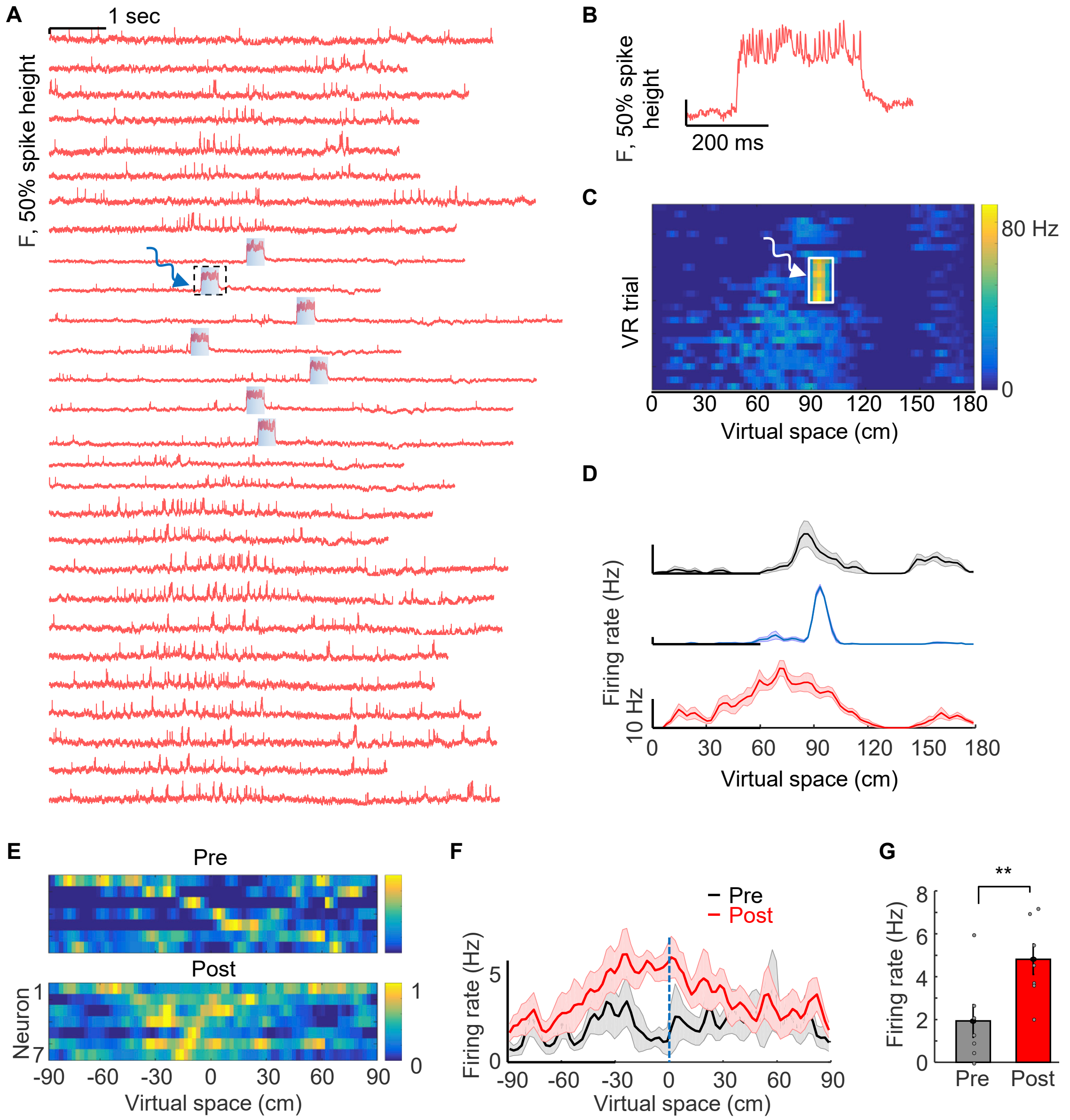

Figure S2. All-optical induction and recording of hippocampal behavioral timescale plasticity, related to Figure 2

(A–D) Example of optogenetic stimulation of pre-existing place cells. (A) Fluorescence traces as a function of time for VR trials pre-, during, and post-optogenetic stimulation. Blue: 300 ms targeted optogenetic stimulation at 90 cm. (B) Magnified view of the boxed region in (A). (C) Firing rate of all VR trials (pre-, during, and post-optogenetic stimulation) across virtual space. White: 300 ms targeted optogenetic stimulation at the 90 cm location. (D) Average firing rate maps pre- (black), during (blue), and post- (red) optogenetic stimulation.

(E–G) Plasticity of optogenetically stimulated cells that did not pass place cell significance test post-optogenetic stimulation (n = 7 cells).

(E) Normalized firing rates.

(F) Average firing rate maps.

(G) Firing rate 10–30 cm before the optically stimulated location significantly increased post-optogenetic stimulation vs. pre-optogenetic stimulation (pre: 1.9 ± 0.8 Hz, post: 4.8 ± 0.7 Hz, p = 0.02, two-sided paired-sample t test).

All error bars and shading: SEM.

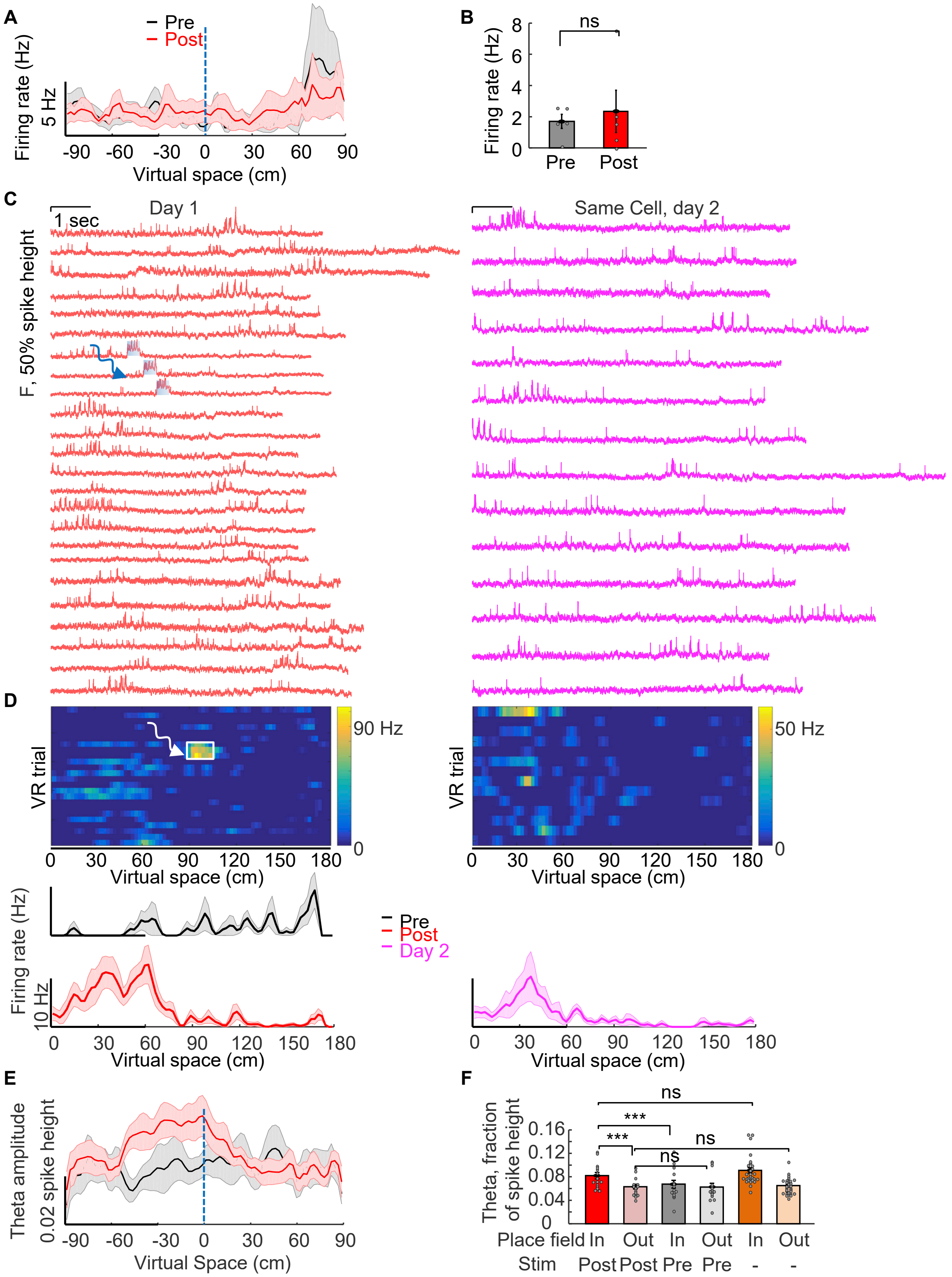

(legend on next page)

CellPress
OPEN ACCESS

Figure S3. Characterization of optically induced plasticity: specificity, stability, and increased theta-oscillation amplitude, related to Figures 2 and 3

(A and B) Control experiments targeting optogenetic stimulation only to single cells while simultaneously imaging surrounding cells. (A) Average firing rate maps of cells that were simultaneously imaged but not optically stimulated. Dashed blue line: stimulation location. (B) Firing rate 10–30 cm before the optically stimulated position did not change for cells not receiving optogenetic stimulation (pre: 1.7 ± 0.5 Hz; post: 2.3 ± 1.4 Hz, p = 0.61, n = 5 cells, two-sided paired-sample t test).

(C and D) Stability of optically induced plasticity. (C) One example cell: fluorescence traces as a function of time for VR trials pre-, during, post-, and 24 h post-optogenetic stimulation. Blue: 300 ms targeted optogenetic stimulation at 90 cm.

(D) Firing rate of all VR trials (pre-, during, post-, and 24 hours post-optogenetic stimulation) across virtual space. White: 300 ms targeted optogenetic stimulation at the 90 cm location. Bottom: average firing rate maps.

(E and F) Optically induced plasticity exhibited enhanced theta-oscillation amplitude. (E) Mean theta-rhythm amplitude (spikes removed and bandpass filtered 6–10 Hz, Hilbert transformed amplitude) of optogenetically stimulated cells aligned at 0 cm. Black: pre-optogenetic stimulation. Red: post-optogenetic stimulation. (F) Optogenetic stimulation did not affect out-of-field theta power (post: 6.3% ± 0.4% of spike height; pre: 6.3% ± 0.6% of spike height, p = 0.89, two-sided paired-sample t test, n = 15 cells), but increased the in-field theta power (post: 8.2% ± 0.6% of spike height, pre: 6.7% ± 0.7% of spike height, p = 0.008, two-sided paired-sample t test). Optogenetically induced place cells had similar in-field and out-of-field theta power as natural place cells (in-field: p = 0.23, out-of-field: p = 0.68, two-tailed t test).

All error bars and shading: SEM.

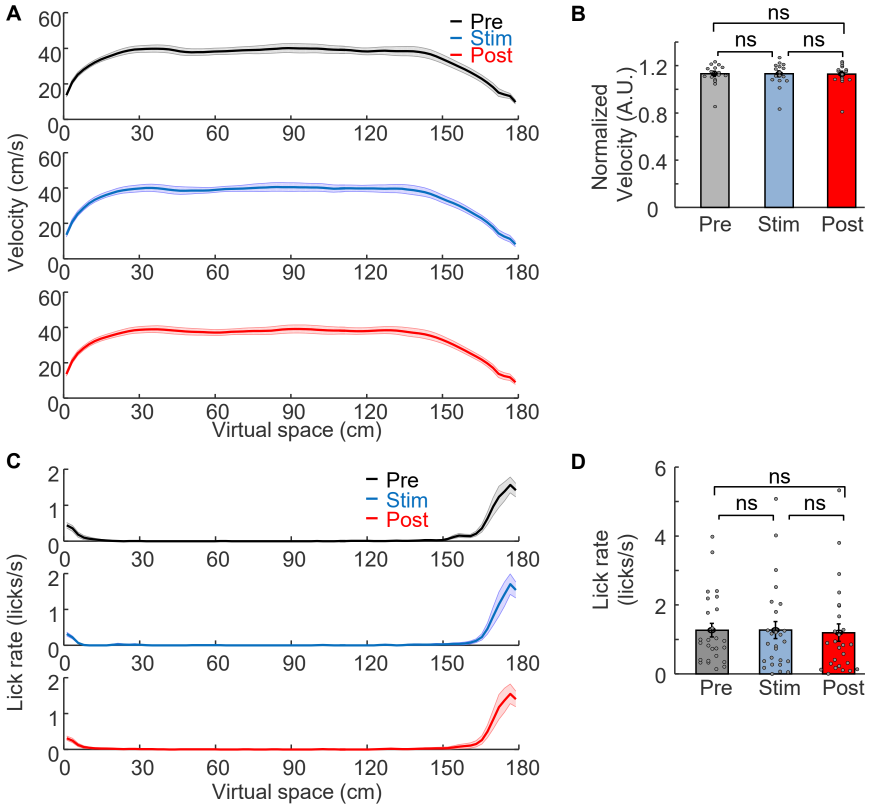

Figure S4. Running and licking behavior did not change with optogenetic induction of plasticity, related to Figure 2

(A) Average running velocity across virtual space during pre, stim, and post epochs.

(B) Average running velocity around stimulation location (90–110 cm) during pre, stim, and post epochs did not change (n = 9 mice, 16 sessions).

(C) Average lick rate across virtual space during pre, stim, and post epochs.

(D) Average licking rate around reward location (170–180 cm) during pre, stim, and post epochs did not change (n = 10 mice, 26 sessions).

All error bars and shading: SEM.

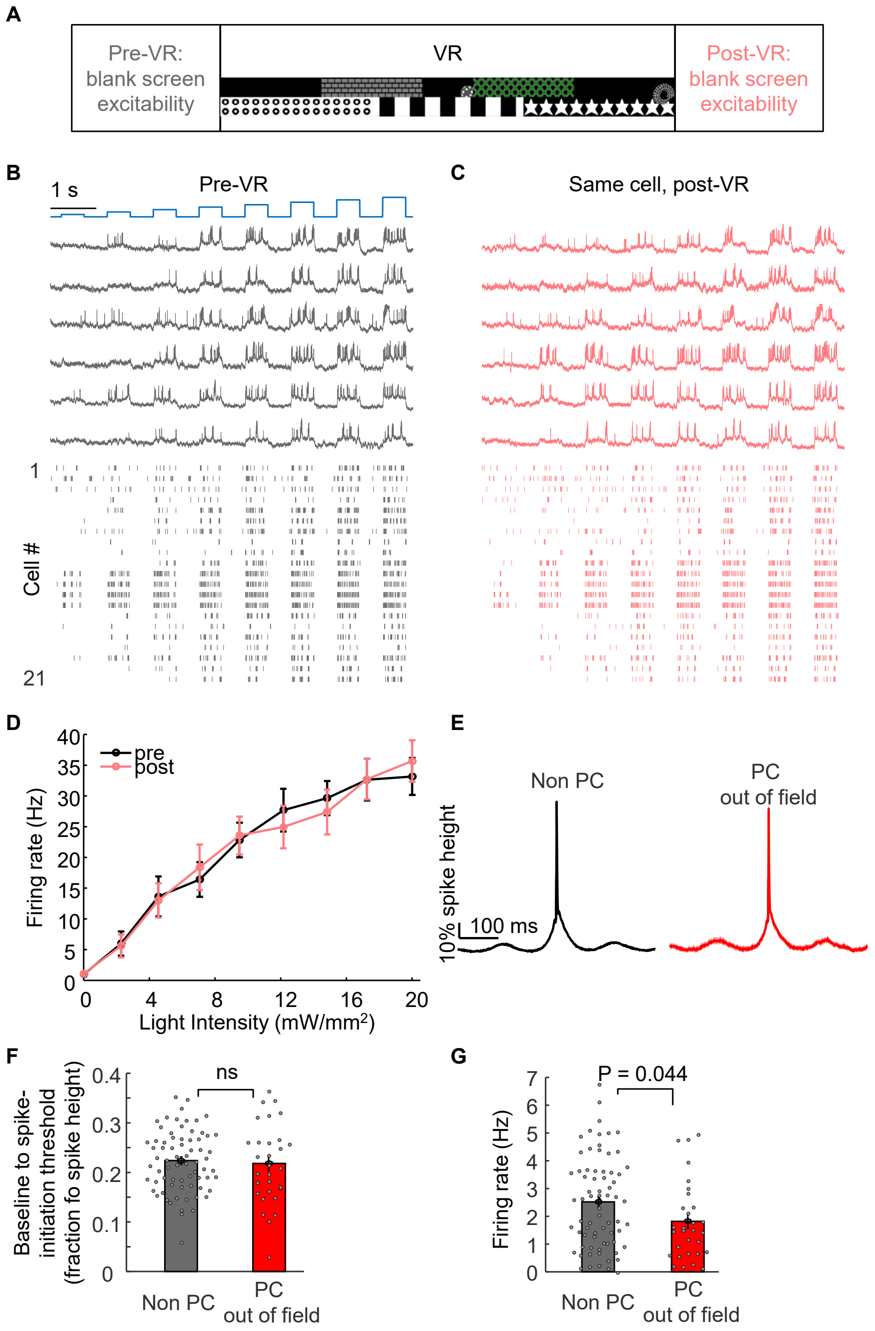

Figure S5. Optically-assessed single-cell excitability parameters without induction of place-field plasticity, related to Figure 4

(A) Excitability was assessed out of the VR environment, before vs. after one VR session without optogenetically induced plasticity.

(B and C) Paired recordings of the same cells. Blue: patterned optogenetic stimulation with steps of blue light (500 ms duration, 0–20 mW/mm^2). Black: example fluorescence of somQuasAr6a during optogenetic stimulation. Red: fluorescence recording of the same cell after the VR session. Bottom: spike raster before and after one VR session without plasticity induction for n = 21 cells.

(D) Mean firing rate as a function of optogenetic stimulus strength (F-I curve) pre- (black) and post- (red) VR behavior.

(E–G) Comparison of excitability between natural place cells and non-place cells (same dataset as in Figure 1). (E) Spike-triggered average waveform of non-place cells (left, n = 71 neurons) and of place cells out of field (right, n = 30 neurons) action potentials. (F) Baseline to spike-initiation threshold did not differ between non-place cells and place cells (non-place cells: 22.4% ± 0.7% of spike height, place cells out-of-field: 21.8% ± 1.5% of spike height, p = 0.69, two-tailed t test). (G) Firing rate of non-place cells was slightly higher than the out-of-field firing rate of place cells (non-place cells: 2.5 ± 0.2 Hz, place cells out-of-field: 1.8 ± 0.3 Hz, p = 0.044, two-tailed t test).

All error bars and shading: SEM.

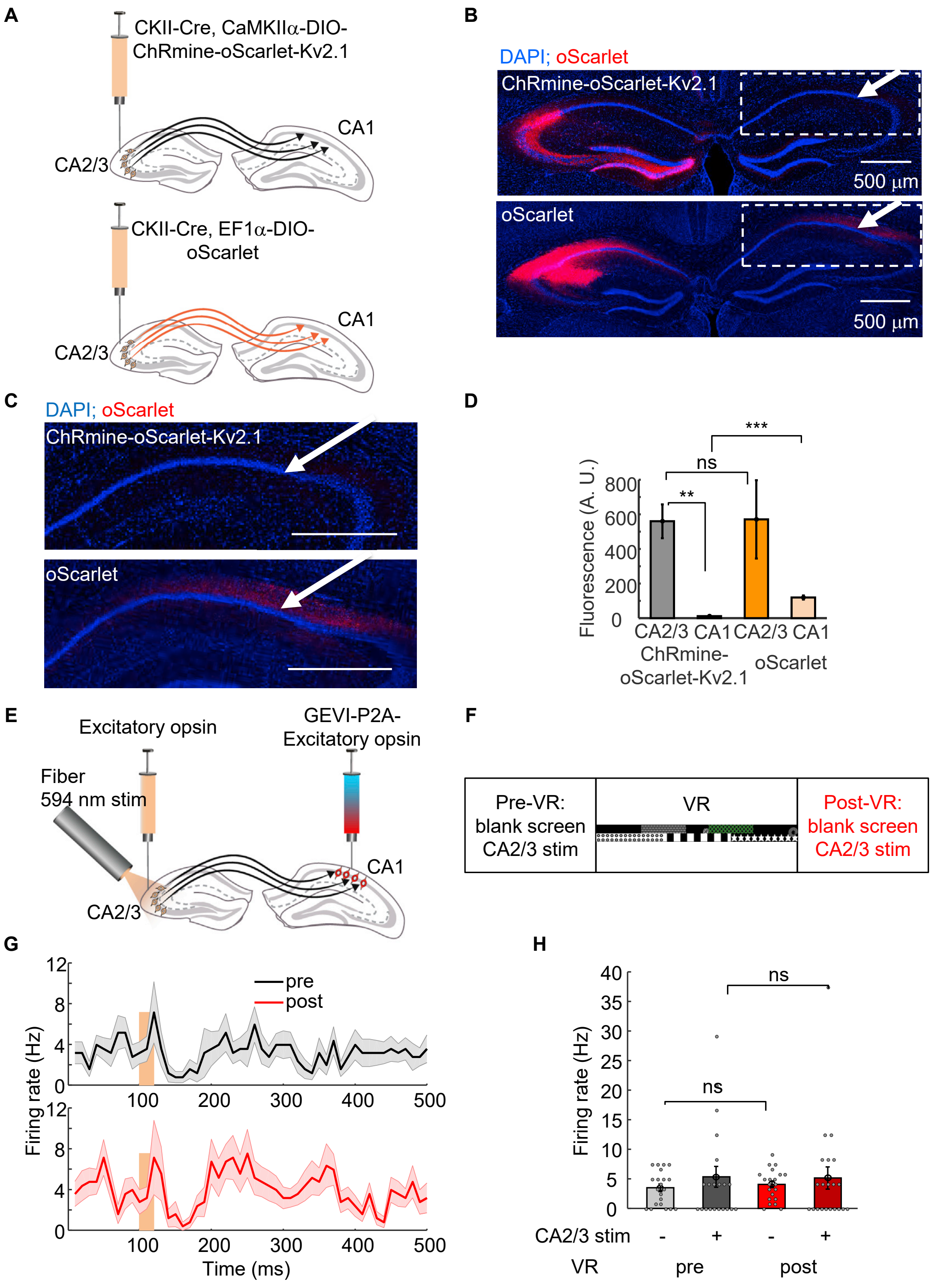

Figure S6. Control conditions for all-optical electrophysiology of CA2/3-to-CA1 synaptic transmission, related to Figures 5, 6, and 7

(A–D) Dense ChRmine-oScarlet-Kv2.1 expression in CA2/3 revealed undetectable axonal fluorescence in contralateral CA1. (A) DIO-ChRmine-oScarlet-Kv2.1 virus (top) and DIO-oScarlet virus (bottom) with high-titer CKII-Cre virus injected in CA2/3. (B) Confocal images of fixed brain slices expressing soma-localized ChRmine-oScarlet-Kv2.1 (top) and cytosolic oScarlet (bottom). Arrows indicate absence (top) and presence (bottom) of oScarlet fluorescence in CA1 area arising from contralateral CA2/3 cells. Contrast in both images is the same. (C) Zoomed views of CA1 areas from boxed regions in B. Scale bars, 500 μm. (D)

(legend continued on next page)

Quantification of fluorescence intensity in CA2/3 and CA1 showing undetectable axonal fluorescence for ChRmine-oScarlet-Kv2.1 (n = 6 slices/2 animals). a.u., arbitrary units.

(E–H) Stability of optically-assessed synaptic transmission without induction of place-field plasticity. Same as Figure 6 but without induction of place-field plasticity. (E) Optical assay of synaptic function between hippocampal CA2/3 and CA1. (F) Synaptic function was assessed out of the VR environment before vs. after one VR session without optogenetically induced plasticity. (G) Mean spike rate during CA2/3 stimulation pre- and post- VR behavior. (H) Quantification of CA2/3 stimulation effect, pre- vs. post- VR behavior. CA2/3 stimulation induced spike rate post-VR behavior was similar to pre-VR behavior, measured over the 1–20 ms window following CA2/3 test pulses onset (pre: 5.4 ± 1.8 Hz, post: 5.2 ± 1.9 Hz, n = 21 cells, p = 0.89, two-sided paired-sample t test). The spontaneous firing rate defined by the 200 ms time window before the CA2/3 test-pulses did not change either (pre: 3.5 ± 0.6 Hz; post: 4.1 ± 0.6 Hz, p = 0.49, two-sided paired-sample t test).

All error bars and shading: SEM.

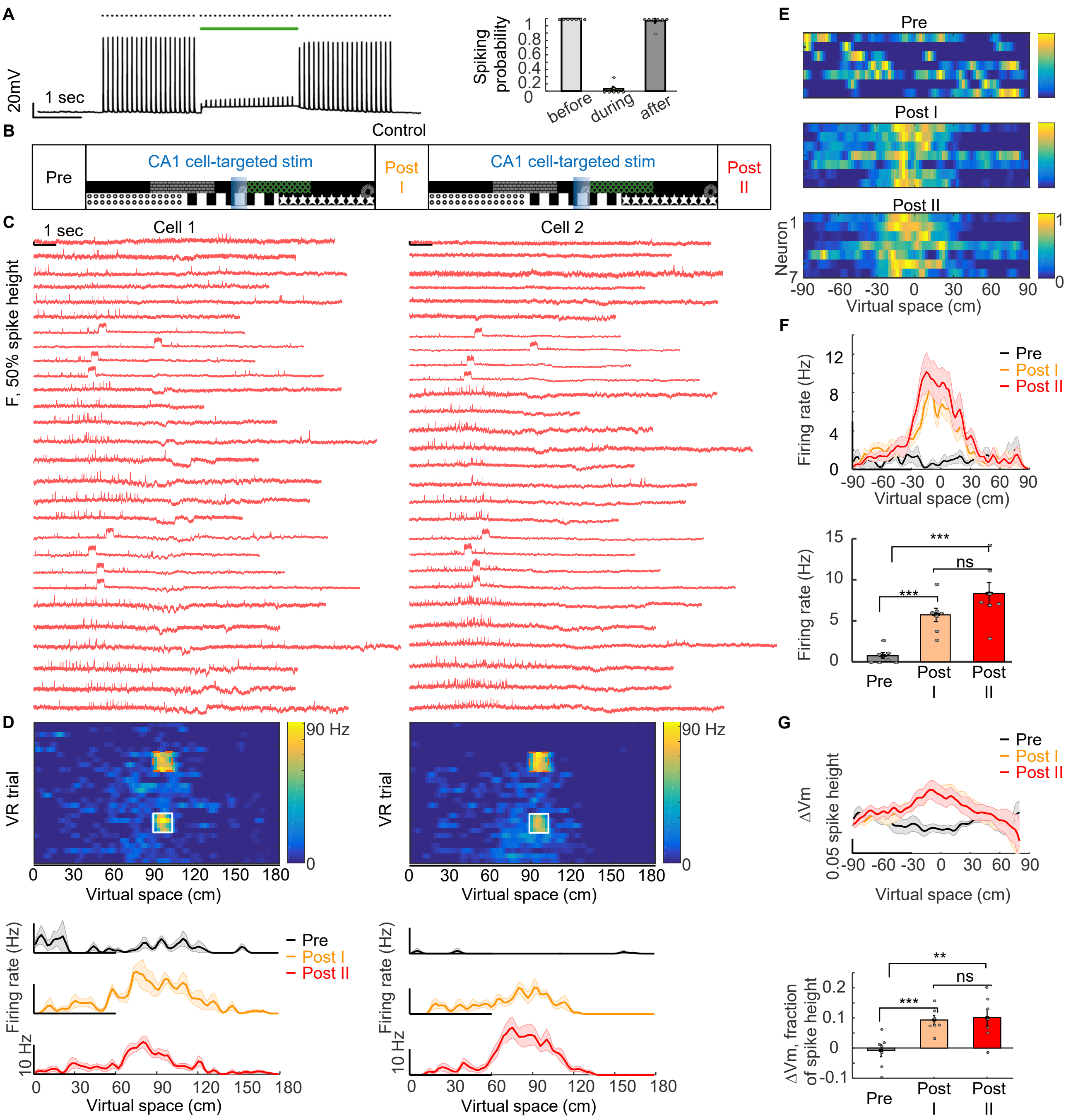

Figure S7. Control conditions for assessing the role of CA2/3 activity for plasticity induction in CA1, related to Figure 7

(A) Whole-cell recordings in acute slices expressing eHcKCR1-3.0. Left, representative voltage trace of a CA2/3 neuron stimulated with pulsed current injections (1 ms pulses, 10 Hz). $V_{rest} = -72$ mV. Electrically evoked spikes were inhibited by green illumination (513 nm, 2 s, 1 mW/mm^2). Right: quantification of spike inhibition (n = 7 cells).

(B–D) Same as Figures 7C–7E) but the control condition replicating the temporal structure of Figure 7 but lacking CA2/3 inhibition.

(E) Control condition: normalized firing rates of all cells (n = 7 cells, 2 mice). Optogenetic stimulation locations were aligned at 0 cm.

(F) Top: average firing rate maps of all cells aligned at 0 cm. Bottom: firing rate (10–30 cm before the optically stimulated location) was significantly increased both during the Post I and Post II epochs compared to the Pre epoch (pre: 0.7 ± 0.4 Hz, post I: 5.7 ± 0.8 Hz, post II: 8.3 ± 1.4 Hz, pre vs. post I, $p = 9.8 \times 10^{-4}$, pre vs. post II, p = 0.003, post I vs. post II, p = 0.1, two-sided paired-sample t test).

(legend continued on next page)

(G) Top: average subthreshold membrane potential of all cells aligned at 0 cm. Bottom: subthreshold membrane potential (10–30 cm before the optically stimulated location) was significantly higher both during the Post I and Post II epochs compared to the Pre epoch (pre: −0.8% ± 2.0% of spike height, post I: 9.4% ± 1.5% of spike height, post II: 10.2% ± 2.8% of spike height, pre vs. post I, p = 0.004, pre vs. post II, p = 0.02, post I vs. post II, p = 0.64, two-sided paired-sample t test).

All error bars and shading: SEM.
